# Myeloid Cells and Sensory Nerves Mediate Peritendinous Adhesion Formation via Prostaglandin E2

**DOI:** 10.1002/advs.202405367

**Published:** 2024-08-29

**Authors:** Xinshu Zhang, Yao Xiao, Zaijin Tao, Yizhe Zhang, Xuan Cheng, Xuanzhe Liu, Yanhao Li, Weiguang Yin, Jian Tian, Shuo Wang, Tianyi Zhang, Xiao Yang, Shen Liu

**Affiliations:** ^1^ Department of Orthopaedics Shanghai Sixth People's Hospital Affiliated to Shanghai Jiao Tong University School of Medicine Shanghai 200233 P. R. China; ^2^ State Key Laboratory of Proteomics Beijing Proteome Research Center National Center for Protein Sciences (Beijing) Beijing Institute of Lifeomics Beijing 102206 P. R. China; ^3^ Department of Orthopaedics Wuxi Ninth People's Hospital Affiliated to Soochow University Wuxi 214062 P. R. China

**Keywords:** myeloid cells, peritendinous adhesion, PGE2, sensory nerves, tendon repair

## Abstract

Peritendinous adhesion that forms after tendon injury substantially limits daily life. The pathology of adhesion involves inflammation and the associated proliferation. However, the current studies on this condition are lacking, previous studies reveal that cyclooxygenase‐2 (COX2) gene inhibitors have anti‐adhesion effects through reducing prostaglandin E2 (PGE2) and the proliferation of fibroblasts, are contrary to the failure in anti‐adhesion through deletion of EP4 (prostaglandin E receptor 4) gene in fibroblasts in mice of another study. In this study, single‐cell RNA sequencing analysis of human and mouse specimens are combined with eight types of conditional knockout mice and further reveal that deletion of COX2 in myeloid cells and deletion of EP4 gene in sensory nerves decrease adhesion and impair the biomechanical properties of repaired tendons. Furthermore, the COX2 inhibitor parecoxib reduces PGE2 but impairs the biomechanical properties of repaired tendons. Interestingly, PGE2 local treatment improves the biomechanical properties of the repaired tendons. These findings clarify the complex role of PGE2 in peritendinous adhesion formation (PAF) and tendon repair.

## Graphical abstract

The deletion of COX2 gene in myeloid cells and the deletion of EP4 gene in sensory nerves decrease (PAF). Myeloid cells and sensory nerves mediate (PAF) via prostaglandin E2.

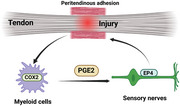

## 1 Introduction

Peritendinous adhesion formation (PAF) is a common complication after tendon injury or tenolysis that limits limb movement.^[^ ^1^ ^]^ The incidence of this condition after tendon injury is reported to be 40%, while the incidence rate of tendon injury is 33.2 injuries/100000 person‐years and brings a heavy economic burden.^[^ ^2^ ^]^ The pathology of PAF involves scar tissue rather than a newly regenerated tendon growing into the injured tendon sites with impaired mechanical properties of tendon.^[^ ^3^ ^]^ PAF usually occurs due to fibroblast proliferation, which is initially triggered by inflammatory factors.^[^ ^1^, ^4^ ^]^ Among these molecules, prostaglandin E2 (PGE2) is a well‐known factor related to inflammation.^[^ ^5^ ^]^ A recent study revealed that the PGE2 level was increased after tendon inflammation which promoted vasodilation and induced a pain hypersensitivity response.^[^ ^6^ ^]^ Several studies have shown that, upstream of PGE2, COX2 (also known as cyclooxygenase‐2) can be inhibited by COX2 inhibitors, which significantly reduce PGE2 and fibroblast proliferation and are associated with in vivo anti‐adhesion effect.^[^ ^7^ ^]^ However, as downstream PGE2 receptors, in vivo deletion of EP4 (prostaglandin E receptor 4) in fibroblasts failed in effective anti‐adhesion.^[^ ^8^ ^]^ If the anti‐adhesion effect is due to inhibition of PGE2 in fibroblasts by COX2 inhibitors, this is contrary to the anti‐adhesion failure in deletion of EP4 in fibroblasts. Therefore, the possible pathology of PGE2 still needs further investigation.

COX2 is considered as a rate‐limiting enzyme in PGE2 synthesis.^[^ ^5^, ^9^ ^]^ For PAF treatment, COX2 inhibitors (including ibuprofen, aspirin, indomethacin, and celecoxib) have been widely used to prevent PAF after tendon injury.^[^ ^10^ ^]^ The possible mechanism of COX2 inhibitors involves anti‐inflammatory and anti‐proliferative effects on fibroblasts via the inhibition of COX2 expression.^[^ ^11^ ^]^ Furthermore, aspirin is a non‐selective COX inhibitor that can decrease adhesion by regulating JNK/STAT‐3 signaling to inhibit inflammation and scar formation.^[^ ^10a^ ^]^ In addition, as a selective COX2 inhibitor rather than a non‐selective COX inhibitor, celecoxib was shown to significantly reduce the proliferation of fibroblasts by inhibiting ERK1/2 phosphorylation.^[^ ^7b^ ^]^ Thus, due to the bypass anti‐adhesion mechanisms of COX2 inhibitors, the molecular anti‐adhesion pathology is more complex than inhibition of COX2 only in fibroblasts.

As PGE2 receptors, EP1, EP2, EP3, and EP4 have many effects on the downstream of PGE2.^[^ ^5^, ^12^ ^]^ However, no studies have investigated the function of EP1, EP2, or EP3 function during the formation of adhesion. The only study on PGE2 receptors involved the deletion of EP4 in fibroblasts. As a result, the PAF was transiently inhibited 2 weeks after modeling in the mice.^[^ ^8^ ^]^ However, the adhesion formation increased again 4 weeks after modeling.^[^ ^8^ ^]^ Although this phenomenon was explained by the increase EP4 level in myofibroblasts, the failed anti‐adhesion formation of deletion of EP4 in fibroblasts was still contrast with the effective anti‐adhesion of COX2 inhibitors through reducing PGE2 and the proliferation of fibroblasts. Therefore, the function of EP1, EP2, EP3, and EP4 in PAF, especially the possible molecular pathways of PGE2 and its origin, need further verification to contribute to drug selection in the clinic.

**Scheme 1:**
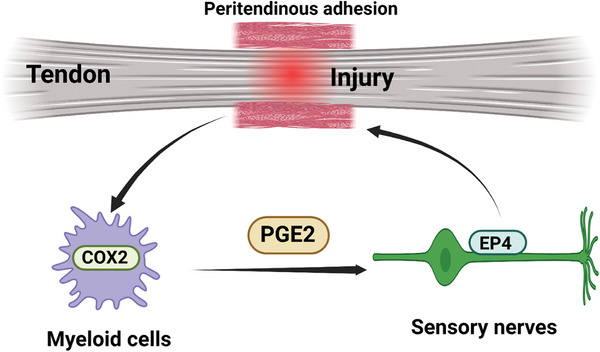
Myeloid cell‐derived PGE2 mediates PAF through EP4 in sensory nerves.

Our previous studies revealed that inflammatory cells and associated effector cells result in PAF through cascade hyperplasia.^[^ ^13^ ^]^ Previous studies showed that sensory nerves participated in tendon healing.^[^ ^14^ ^]^ However, the functional origin and effector cells of PGE2 and the functions of sensory nerves are still unknown. Thereafter, in this study, the in vivo function of PGE2 in PAF is verified by using multiple up‐down pathway transgenic mice. Furthermore, single‐cell RNA sequencing (scRNA‐seq) of human and mouse specimens is performed to identify the possible pathway of PGE2. In addition, the effect of PGE2 on tendon repair is investigated. We finally reveal that myeloid cells secrete PGE2 and activate EP4 in sensory nerves. We prove that sensory nerves are related to PGE2, sensory nerves participate in PAF and tendon healing which increase peritendinous adhesion (**Scheme** **1**).

## 2 Results

### 2.1 scRNA‐Seq Analysis of Human Tendon Adhesion Tissues

Based on our previous studies, we had performed scRNA‐seq analysis of human tendon adhesion tissues.^[^ ^13c^ ^]^ We had identified seven major cell types: Macrophages highly expressed CD68 molecule (*CD68*) and CD163 molecule (*CD163*).^[^ ^15^ ^]^ Fibroblasts were characterized by high expression of lumican (*LUM*) and decorin (*DCN*).^[^ ^16^ ^]^ Endothelial cells were marked by platelet and endothelial cell adhesion molecule 1 (*PECAM1*) and Von Willebrand factor (*VWF*).^[^ ^16^, ^17^ ^]^ Neutrophils were identified by the high expression of colony stimulating factor 3 receptor (*CSF3R*) and Fc gamma receptor IIIb (*FCGR3B*).^[^ ^18^ ^]^ B cells highly expressed CD79a molecule (*CD79A*) and membrane spanning 4‐domains A1 (*MS4A1*).^[^ ^19^ ^]^ T cells were marked by CD3 delta subunit of T‐cell receptor complex (*CD3D*) and CD3 epsilon subunit of T‐cell receptor complex (*CD3E*).^[^ ^20^ ^]^ Mast cells were identified by high expression of membrane spanning 4‐domains A2 (*MS4A2*) and tryptase beta 2 (*TPSB2*) (Figure S1a, Supporting Information).^[^ ^21^ ^]^

**Figure 1:**
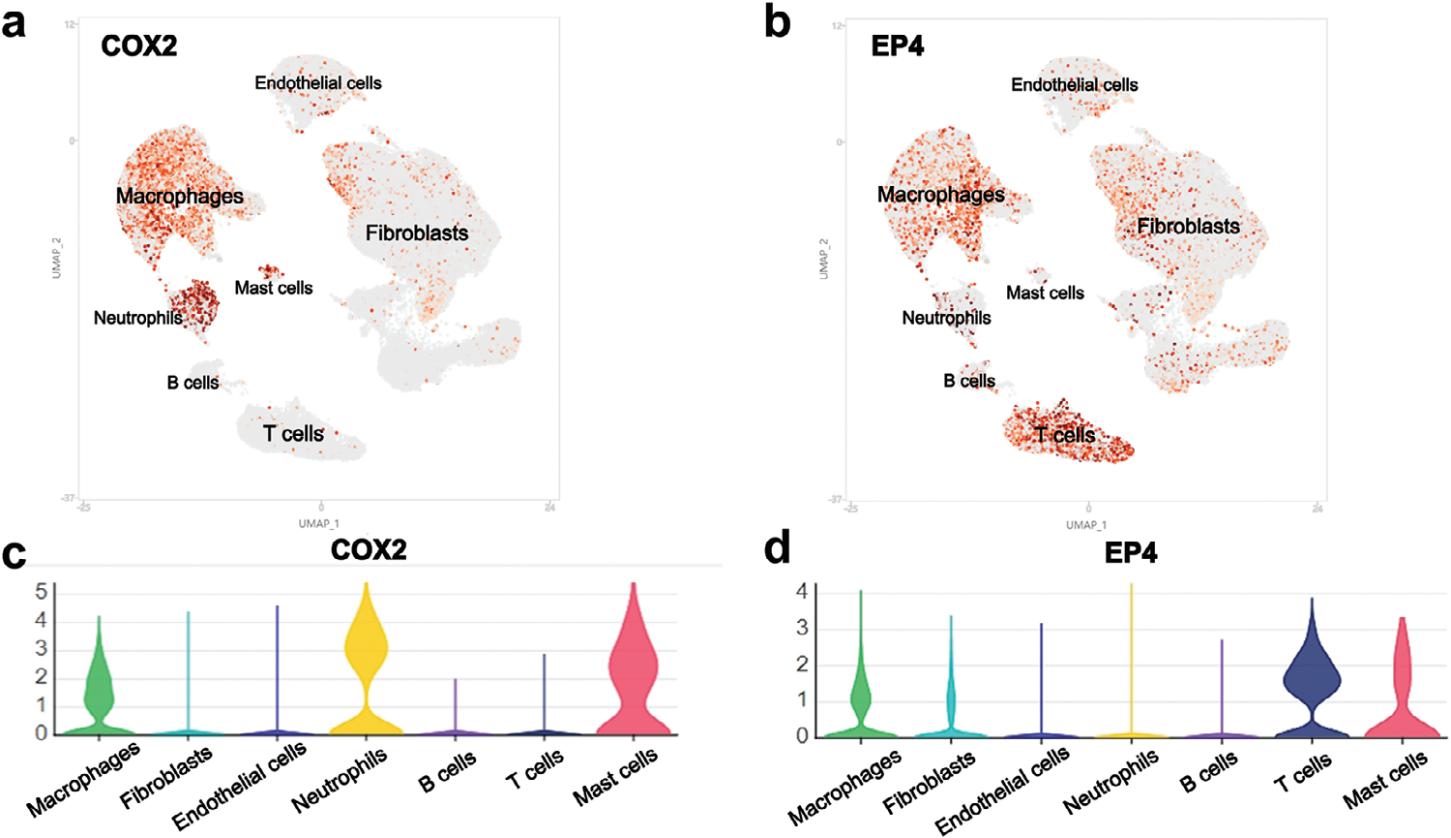
scRNA‐seq of human peritendinous adhesion tissues. a) UMAP plots showing the expression of COX2. b) UMAP plots showing the expression of EP4. c) Violin plots showing COX2 expression in each cluster. d) Violin plots showing EP4 expression in each cluster. UMAP, uniform manifold approximation and projection.

Uniform manifold approximation and projection (UMAP) and violin plots revealed that COX2 was mostly expressed in myeloid cells (such as macrophages, neutrophils, and mast cells), previous studies indicated that myeloid cells included macrophages, neutrophils, and mast cells (**Figure** **1**a,c).^[^ ^22^ ^]^ EP1, EP2, and EP3 were rarely expressed in human tendon adhesion (Figure S1b, Supporting Information). UMAP and violin plots revealed that EP4 was expressed in myeloid cells (such as macrophages, neutrophils, and mast cells), fibroblasts, and T cells (Figure 1b,d). These findings suggested that EP4, rather than EP1, EP2, or EP3, was involved in human PAF.

### 2.2 scRNA‐Seq Analysis of Mouse Tendon Adhesion Tissues

**Figure 2:**
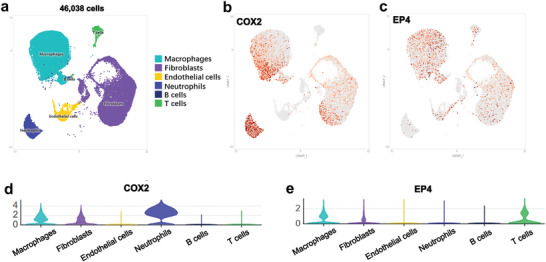
scRNA‐seq of mouse peritendinous adhesion tissues. a) UMAP plots of 46308 cells in mouse peritendinous adhesion tissues. b) UMAP plots showing the expression of COX2. c) UMAP plots showing the expression of EP4. d) Violin plots showing COX2 expression in each cluster. e) Violin plots showing EP4 expression in each cluster. UMAP, uniform manifold approximation and projection.

We performed scRNA‐seq analysis of mouse tendon adhesion tissues. More than 46000 cells were profiled (**Figure** **2**a). Six major cell clusters were identified: macrophages, fibroblasts, endothelial cells, neutrophils, B cells, and T cells (Figure 2a). Macrophages were identified by the expression of *Cd68* and mannose receptor C‐type 1 (*Mrc1*).^[^ ^15a^ ^]^ Fibroblasts were identified by the expression of *Lum* and *Dcn*.^[^ ^16^ ^]^ Endothelial cells were identified by the high expression of *Pecam1* and *Vwf*.^[^ ^17^, ^23^ ^]^ Neutrophils, B cells and T cells were identified by the expression of *Csf3r*, *Cd79a* and *Cd3d*, respectively (Figure S2a,b, Supporting Information).^[^ ^18^, ^19^, ^20^ ^]^ Macrophages and fibroblasts were the major types of cells in mouse tendon adhesion tissues (Figure 2a).

UMAP and violin plots revealed that COX2 was mostly expressed in myeloid cells (such as macrophages and neutrophils) and fibroblasts (Figure 2b,d). EP1, EP2, and EP3 were rarely expressed in mouse tendon adhesion (Figure S2a, Supporting Information). UMAP and violin plots revealed that EP4 was expressed in macrophages, fibroblasts, and T cells (Figure 2c,e). These findings suggested that EP4, rather than EP1, EP2, or EP3, was involved in mouse PAF.

### 2.3 PGE2 Secreted by Myeloid Cells and Fibroblasts Mediates Mouse PAF and Tendon Repair

Based on the scRNA‐seq analysis of human and mouse peritendinous adhesion tissues, we generated mice in which COX2 was deleted in myeloid cells (including macrophages and neutrophils) (*Lysm‐cre:COX2^flox/flox^*) by crossing *Lysm‐cre* mice with *COX2^flox/flox^* (*COX2^f/f^*) mice, mice in which COX2 was deleted in fibroblasts (*S100a4‐cre:COX2^flox/flox^*) by crossing *S100a4‐cre* mice with *COX2^flox/flox^* (*COX2^f/f^*) mice and mice in which COX2 was deleted in myofibroblasts (*α‐SMA‐cre:COX2^flox/flox^*) by crossing *α‐SMA‐cre* mice with *COX2^flox/flox^* (*COX2^f/f^*) mice.

**Figure 3:**
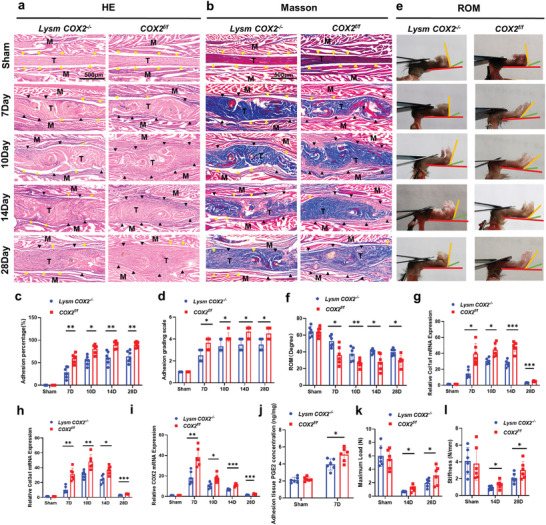
Deletion of COX2 in Lysm^+^ cells reduced PAF but impaired the biomechanical properties of repaired tendons. *Lysm‐cre: COX2^flox/flox^* (*Lysm COX2^−/−^*) mice were generated by crossing *Lysm‐cre* mice with *COX2^flox/flox^* (*COX2^f/f^*) mice. a,b) Representative images of H&E (a) and Masson (b) staining of the peritendinous tissues from *Lysm COX2^−/‐^* and *COX2^f/f^* mice at 7, 10, 14, and 28 days after modeling. Yellow arrowheads indicate the space between the tendon and surrounding tissues. Black arrowheads indicate the space occupied by adhesion tissues. Scale bar, 500 µm. c,d) Adhesion percentage (c) and adhesion grading scale (d) of the peritendinous tissues from *Lysm COX2^−/‐^* and *COX2^f/f^* mice at 7, 10, 14 and 28 days after modeling. e,f) Investigation analysis (e) and quantitative analysis (f) of ROM. g,h,i) Relative mRNA expression of *COL1A1* (g), *COL3A1* (h) and *COX2* (i) of the peritendinous tissues from *Lysm COX2^−/‐^* and *COX2^f/f^* mice at 7, 10, 14, and 28 days after modeling. j) PGE2 levels (ng PGE2/mg protein) of the peritendinous tissues from *Lysm COX2^−/‐^* and *COX2^f/f^* mice at 7 days after modeling. k,l) Maximum load (k) and stiffness (l) of repaired tendons from *Lysm COX2^−/‐^* and *COX2^f/f^* mice at 14 and 28 days after modeling. ^*^ indicates *p* < 0.05. ^**^ indicates *p* < 0.01. ^***^ indicates *p* < 0.001. *n* = 6 per group (c‐l). M, muscle; T, tendon; D, day; ROM, range of motion.

**Figure 4:**
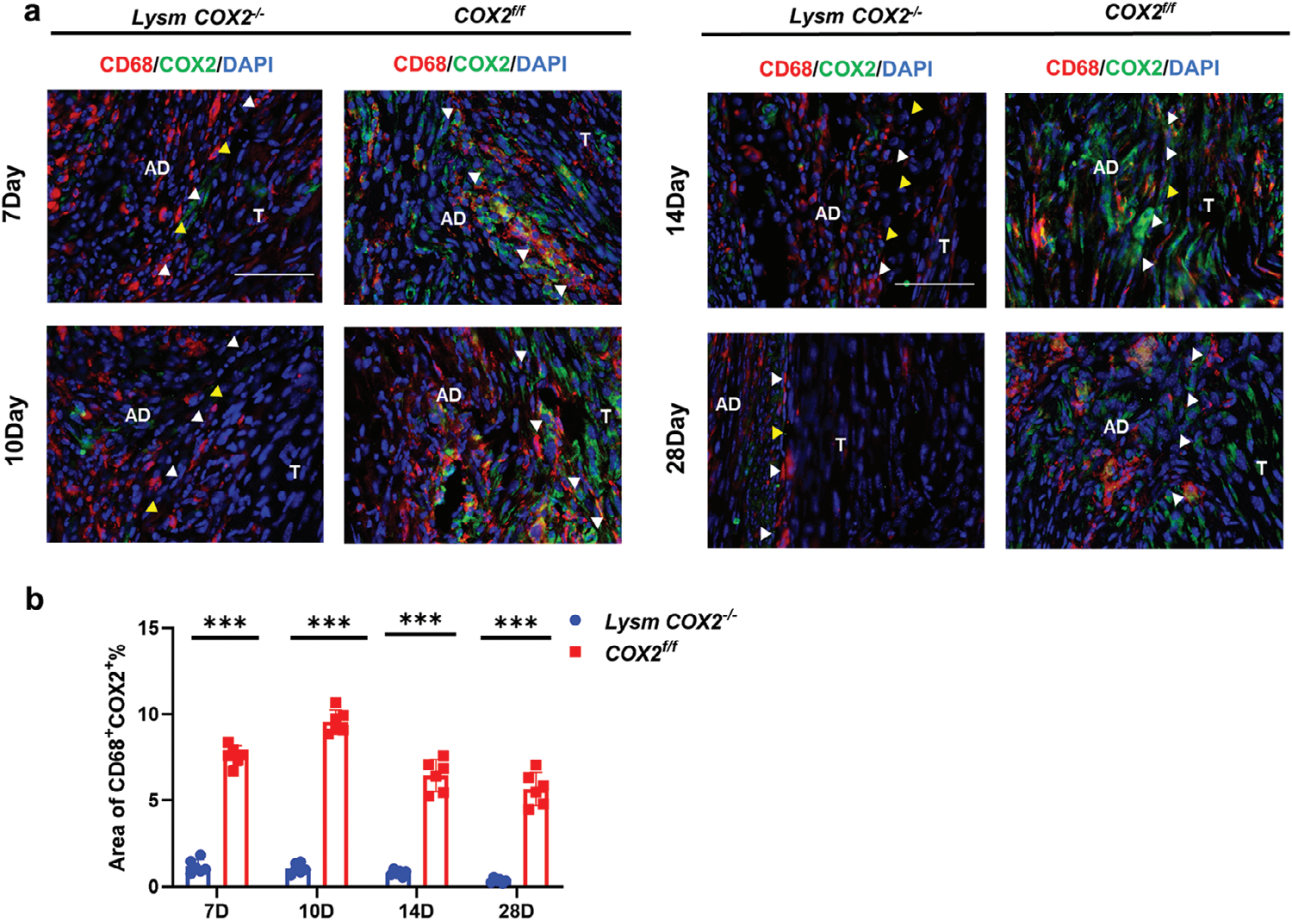
CD68^+^ COX2^+^ cells and peritendinous adhesion decreased in *Lysm COX2^−/^* ^−^ mice. a) Immunofluorescence staining of CD68^+^ (red) COX2^+^ (green) cells in peritendinous adhesion tissues in *Lysm COX2^−/^* ^−^ and *COX2^f/f^* mice 7, 10, 14, and 28 days after modeling. Blue, DAPI. Yellow arrowheads indicate the space between the tendon and surrounding tissues. White arrowheads indicate the space occupied by adhesion tissues. Scale bar, 200 µm. D, day; AD, adhesion; T, tendon. b) Quantitative analysis of the CD68^+^COX2^+^ area in peritendinous adhesion tissues. ^***^ indicates *P*< 0.001. *n* = 6 per group (b).

The flexor digitorum longus tendons of these mice and their *COX2^f/f^* littermates were subjected to surgery to model tendon injury (Figure S9, Supporting Information). H&E and Masson staining of peritendinous adhesion revealed decreased adhesion in *Lysm‐cre: COX2^flox/flox^* (*Lysm COX2^−/−^*) mice and *S100a4‐cre: COX2^flox/flox^* (*S100a4 COX2^−/−^*) mice 7, 10, 14 and 28 days after modeling (**Figure** **3**a,b; Figure S3a,b, Supporting Information). *α‐SMA‐cre:COX2^flox/flox^* (*α‐SMA COX2^−/−^*) mice were similar to *COX2^f/f^* mice (Figure S4a,b, Supporting Information). The percentage of the adhesion area and adhesion grading scale were evaluated and showed that *Lysm COX2^−/‐^* mice and *S100a4 COX2^−/−^* mice had decreased adhesion area on Days 7, 10, 14 and 28 after modeling (Figure 3c,d; Figures S3c,d and S10, Supporting Information). We evaluated the range of motion (ROM) of these mice. *Lysm COX2^−/−^* mice and *S100a4 COX2^−/−^* mice exhibited significant improvements on Days 7, 10, 14 and 28 after modeling (Figure 3e,f; Figure S3e,f, Supporting Information). The percentage of the adhesion area, adhesion grading scale, and ROM of *α‐SMA COX2^−/−^* mice were not significantly different from those of *COX2^f/f^* mice (Figure S4c–f, Supporting Information). Similarly, the mRNA expression of *Col1a1*, *Col3a1*, and *COX2* in peritendinous adhesion tissues was decreased in *Lysm COX2^−/−^* mice and *S100a4 COX2^−/^* mice 7, 10, 14 and 28 days after modeling (Figure 3g–i; Figure S3g–i, Supporting Information). No significant difference in *Col1a1* or *Col3a1* mRNA expression was observed in *α‐SMA COX2^−/−^* mice (Figure S4g,h, Supporting Information). Enzyme‐linked immunosorbent assay (ELISA) was used to analyze mouse peritendinous adhesion tissues and revealed that the PGE2 levels in *Lysm COX2^−/−^* mice and *S100a4 COX2^−/−^* mice were lower than those in *COX2^f/f^* mice on Day 7 after modeling (Figure 3j; Figure S3j, Supporting Information). Compared with those of *COX2^f/f^* mice, the maximum load and stiffness of repaired tendons in *Lysm COX2^−/−^* mice and *S100a4 COX2^−/−^* mice were reduced on Days 14 and 28 after modeling (Figure 3k,l; Figure S3k,l, Supporting Information). These findings indicated that the biomechanical properties of the repaired tendons of *Lysm COX2^−/−^* mice and *S100a4 COX2^−/−^* mice were impaired. No significant difference in the maximum load and stiffness of repaired tendons was observed in *α‐SMA COX2^−/−^* mice (Figure S4i,j, Supporting Information). Immunofluorescent staining of CD68 (the marker of myeloid cells) and COX2 in peritendinous adhesion from *Lysm COX2^−/−^* and *COX2^f/f^* mice revealed a decrease in the CD68^+^COX2^+^ area and fewer adhesion on Days 7, 10, 14 and 28 after modeling in *Lysm COX2^−/−^* mice (**Figure** **4**a,b). Combined with the scRNA‐seq results, the UMAP plot of α‐SMA (actin alpha 2, the marker of myofibroblasts) and COX2 showed little co‐expression, which may explain why the peritendinous adhesion of *α‐SMA COX2^−/−^* mice were not reduced (Figure 2b; Figure S2a, Supporting Information).^[^ ^16^ ^]^

These results suggested that PGE2 was secreted by myeloid cells and fibroblasts during mouse tendon healing and that the COX2 gene in myofibroblasts was not critical for PGE2 secretion. A reduction in PGE2 levels may decrease PAF but impair the biomechanical properties of repaired tendons.

### 2.4 PGE2 Activates EP4 in Sensory Nerves to Mediate PAF and Tendon Repair

A recent study revealed that PGE2 activates EP4 in sensory nerves to regulate bone formation which indicated that EP4 in sensory nerves was involved in the COX2/PGE2/EP4 pathway.^[^ ^24^ ^]^ Combined with our scRNA‐seq results, we generated mice in which EP4 was deleted in myeloid cells (including macrophages and neutrophils) (*Lysm‐cre:EP4^flox/flox^*) by crossing *Lysm‐cre* mice with *EP4^flox/flox^* (*EP4^f/f^*) mice. Crossing *S100a4‐cre* mice with *EP4^flox/flox^* (*EP4^f/f^*) mice to generate mice in which EP4 was deleted in fibroblasts (*S100a4‐cre:EP4^flox/flox^*). Crossing *α‐SMA‐cre* mice with *EP4^flox/flox^* (*EP4^f/f^*) mice to generate mice in which EP4 was deleted in myofibroblasts (*α‐SMA‐cre:EP4^flox/flox^*). Crossing *Prrx1‐cre* mice with *EP4^flox/flox^* (*EP4^f/f^*) mice to generate mice in which EP4 was deleted in stem cells (*Prrx1‐cre:EP4^flox/flox^*). To generate mice in which EP4 was deleted in sensory nerves (*Advillin‐cre ^ERT2^:EP4^flox/flox^*) by crossing *Advillin‐cre^ERT2^* mice with *EP4^flox/flox^* (*EP4^f/f^*) mice. Six weeks before modeling, *Advillin‐cre^ERT2^:EP4^flox/flox^* mice were intraperitoneally injected with 100 mg kg^−1^ body weight tamoxifen three times per week.

**Figure 5:**
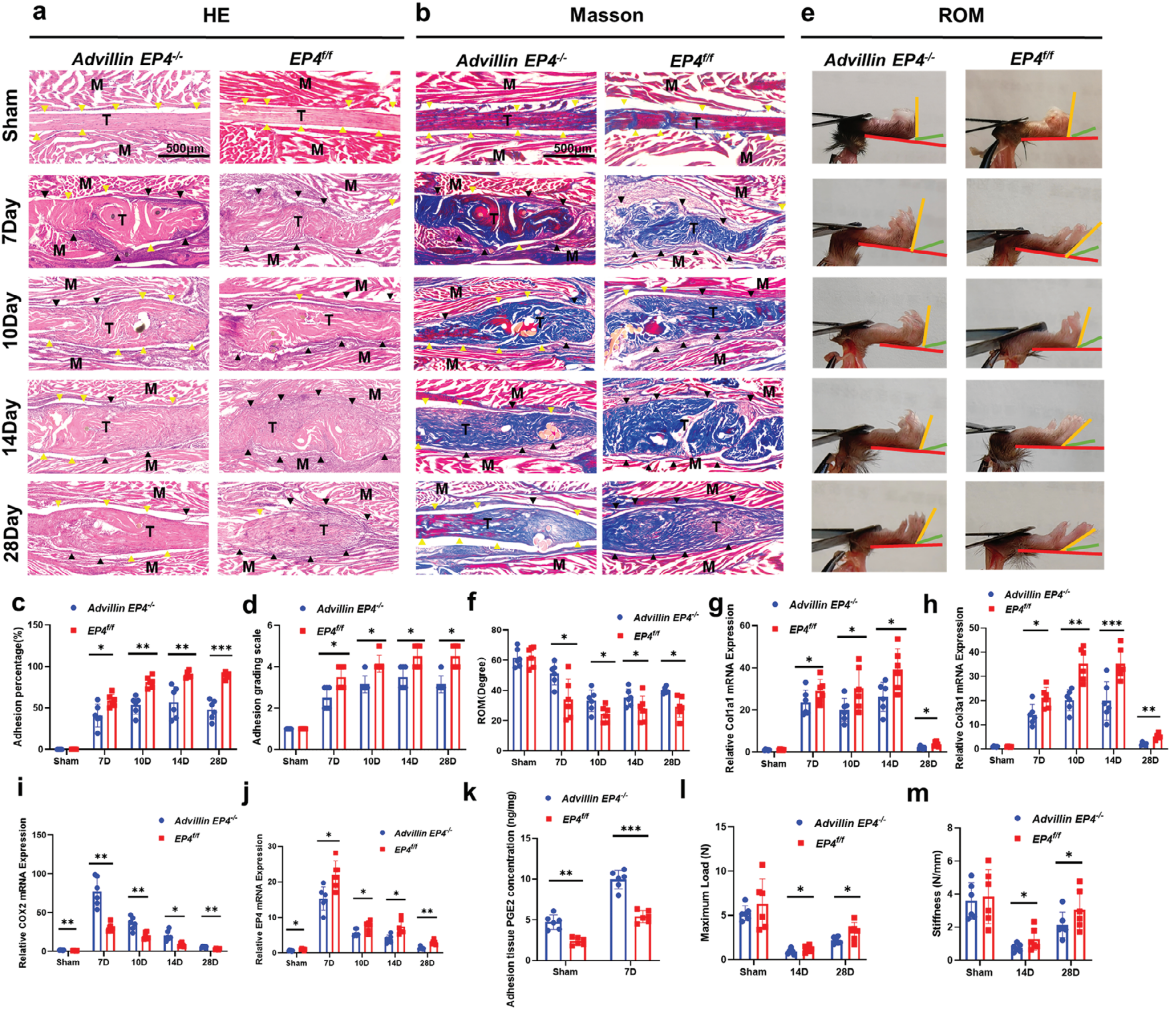
Deletion of EP4 in sensory nerves attenuated PAF but impaired the biomechanical properties of repaired tendons. Tamoxifen‐inducible *Advillin‐cre^ERT2^: EP4^flox/flox^* (*Advillin EP4^−/−^*) mice were generated by breeding *Advillin‐cre^ERT2^* mice with *EP4^flox/flox^* (*EP4^f/f^*) mice. Six weeks before modeling, *Advillin‐cre ^ERT2^:EP4^flox/flox^* mice were intraperitoneally injected with 100 mg kg^−1^ body weight tamoxifen three times per week. a,b) Representative images of H&E (a) and Masson (b) staining of the peritendinous tissues from *Advillin EP4^−/‐^* and *EP4^f/f^* mice at 7, 10, 14, and 28 days after modeling. Yellow arrowheads indicate the space between the tendon and surrounding tissues. Black arrowheads indicate the space occupied by adhesion tissues. Scale bar, 500 µm. c,d) Adhesion percentage (c) and adhesion grading scale (d) of the peritendinous tissues from *Advillin EP4^−/‐^* and *EP4^f/f^* mice at 7, 10, 14, and 28 days after modeling. e, f Investigation analysis (e) and quantitative analysis (f) of ROM. g,h,i,j) Relative mRNA expression of *COL1A1* (g), *COL3A1* (h), *COX2* (i) and *EP4* (j) of the peritendinous tissues from *Advillin EP4^−/‐^* and *EP4^f/f^* mice at 7, 10, 14, and 28 days after modeling. k) PGE2 levels (ng PGE2/mg protein) of the peritendinous tissues from *Advillin EP4^−/‐^* and *EP4^f/f^* mice at 7 days after modeling. l,m) Maximum load (l) and stiffness (m) of repaired tendons from *Advillin EP4^−/‐^* and *EP4^f/f^* mice at 14 and 28 days after modeling. ^*^ indicates *p* < 0.05. ^**^ indicates *p* < 0.01. ^***^ indicates *p* < 0.001. *n* = 6 per group (c‐m). M, muscle; T, tendon; D, day; ROM, range of motion.

**Figure 6:**
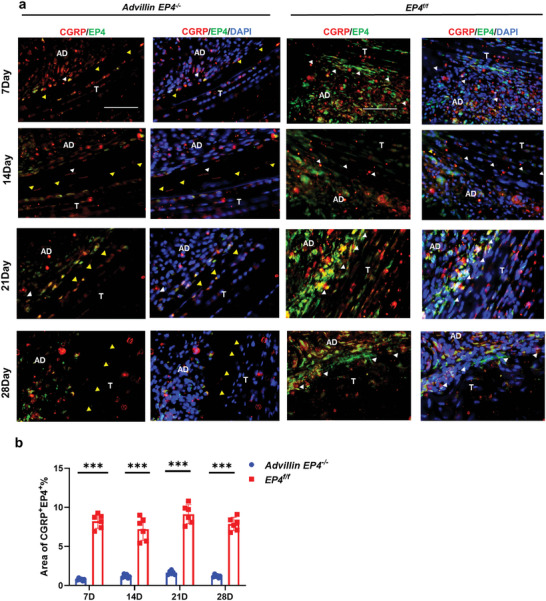
EP4 expression and peritendinous adhesion decreased in *Advillin EP4^−/−^* mice. a) Immunofluorescence staining of CGRP^+^ (red) EP4^+^ (green) cells in peritendinous adhesion tissues in *Advillin EP4^−/^* ^−^ and *EP4^f/f^* mice 7, 14, 21, and 28 days after modeling. Blue, DAPI. Yellow arrowheads indicate the space between the tendon and surrounding tissues. White arrowheads indicate the space occupied by adhesion tissues. Scale bar, 200 µm. b) Quantitative analysis of the CGRP^+^EP4^+^ area in peritendinous adhesion tissues. ^***^ indicates *P*< 0.001. *n* = 6 per group (b). D, day; AD, adhesion; T, tendon.

The flexor digitorum longus tendons of these mice and their *EP4^f/f^* littermates were subjected to surgery (Figure S9, Supporting Information). H&E and Masson staining showed that *Advillin‐cre^ERT2^:EP4^flox/flox^* (*Advillin EP4^−/−^*) mice had fewer peritendinous adhesion on Days 7, 10, 14, and 28 after modeling than *EP4^f/f^* mice (**Figure** **5**a,b). No significant difference in H&E or Masson staining were detected in *Lysm‐cre:EP4^flox/flox^* (*Lysm EP4^−/−^*) mice, *S100a4‐cre:EP4^flox/flox^* (*S100a4 EP4^−/−^*) mice*, Prrx1‐cre:EP4^flox/flox^* (*Prrx1 EP4^−/−^*) mice or *α‐SMA‐cre:EP4^flox/flox^* (*α‐SMA EP4^−/−^*) mice (Figures S5a,b, S6a,b, S7a,b, and Figure S8a,b, Supporting Information). The percentage of the adhesion area and adhesion grading scale were reduced in *Advillin EP4^−/−^* mice on Days 7, 10, 14, and 28 after modeling (Figure 5c,d). The decrease in peritendinous adhesion in the other mice was not significant (Figures S5c,d, S6c,d, S7c,d, and S8c,d, Supporting Information). We evaluated the ROM of these mice, and only *Advillin EP4^−/‐^* mice exhibited a significant increase in ROM on Days 7, 10, 14, and 28 after modeling (Figure 5e,f). The ROM did not significantly change in the other *EP4^−/ −^* mice (Figures S5e,f, S6e,f, S7e,f, and S8e,f, Supporting Information). In the peritendinous adhesion tissues of *Advillin EP4^−/‐^* mice, the mRNA expression of *Col1a1*, *Col3a1*, and *EP4* decreased on Days 7, 10, 14, and 28 after modeling (Figure 5g,h,j). The mRNA expression of *COX2* increased, indicating that COX2 had a compensatory increase in response to the deletion of EP4 in sensory nerves (Figure 5i). The changes in *Col1a1* and *Col3a1* mRNA expression in the other mice were not significant, and only *Lysm EP4^−/−^* mice exhibited a decrease in *Col1a1* mRNA expression on Days 10 and 14 after modeling (Figures S5g,h, S6g,h, S7g,h, and S8g,h). ELISA was used to analyze peritendinous adhesion tissues of mice and revealed that the PGE2 levels in *Advillin EP4^−/‐^* mice were increased in a compensatory manner on Days 7 after modeling, but the maximum load and stiffness of the repaired tendons in *Advillin EP4^−/−^* mice on Days 14 and 28 after modeling were lower than *EP4^f/f^* mice (Figure 5k–m). Immunofluorescent staining of calcitonin gene‐related peptide (CGRP, a marker of sensory nerves) and EP4 in the peritendinous adhesion of *Advillin EP4^−/−^* and *EP4^f/f^* mice revealed a decrease in the CGRP^+^EP4^+^ area in *Advillin EP4^−/−^* mice on Days 7, 14, 21, and 28 after modeling (**Figure** **6**a,b).^[^ ^24^ ^]^

These results suggested that PGE2 activates EP4 in sensory nerves to mediate PAF and tendon repair. Deletion of EP4 in sensory nerves decreased PAF but impaired tendon biomechanical properties.

### 2.5 The COX2 Inhibitor Parecoxib Reduces PAF but Impairs the Biomechanical Properties of Repaired Tendons

**Figure 7:**
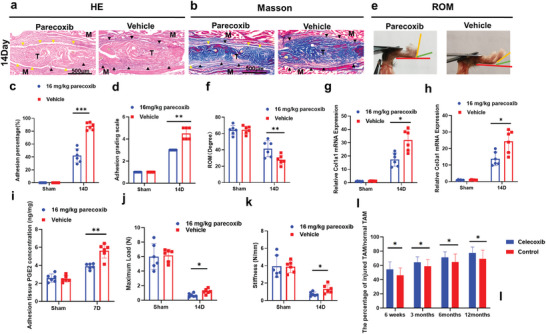
Parecoxib attenuated PAF but impaired the biomechanical properties of repaired tendons. C57BL/6J WT mice were treated with parecoxib (16 mg k^−1^g per day) or vehicle (saline). a,b) Representative images of H&E (a) and Masson (b) staining of the peritendinous tissues from WT mice treated with parecoxib or vehicle at 14 days after modeling. Yellow arrowheads indicate the space between the tendon and surrounding tissues. Black arrowheads indicate the space occupied by adhesion tissues. Scale bar, 500 µm. c,d) Adhesion percentage (c) and adhesion grading scale (d) of the peritendinous tissues from WT mice treated with parecoxib or vehicle at 14 days after modeling. e,f) Investigation analysis (e) and quantitative analysis (f) of ROM. g,h) Relative mRNA expression of *COL1A1* (g) and *COL3A1* (h) of the peritendinous tissues from WT mice treated with parecoxib or vehicle at 14 days after modeling. i) PGE2 levels (ng PGE2/mg protein) of the peritendinous tissues from WT mice treated with parecoxib or vehicle at 7 days after modeling. j,k) Maximum load (j) and stiffness (k) of repaired tendons from WT mice treated with parecoxib or vehicle at 14 days after modeling. l) The percentage of injured TAM/normal TAM in the celecoxib treated patients and untreated patients (controls) with peritendinous adhesion, ^*^ indicates *p* < 0.05. ^**^ indicates *p* < 0.01. ^***^ indicates *p* < 0.001. *n* = 6 per group (c–k). n = 20 per group (l). M, muscle; T, tendon; D, day; ROM, range of motion; TAM, Total Active Motion.

COX2 inhibitors can inhibit the production of PGE2 and reduce pain and inflammation and are widely used to treat tendon injury.^[^ ^25^ ^]^ However, a recent study revealed that COX2 inhibitors may impair tendon healing.^[^ ^25^, ^26^ ^]^ Our previous study suggested that deletion of COX2 in myeloid cells induced a lower level of PGE2 and impaired the biomechanical properties of tendons. Therefore, we treated C57BL/6J wild‐type (WT) mice with parecoxib (16 mg kg^−1^ per day) after tendon surgery. The mice were administered 16 mg kg^−1^ parecoxib every day via intraperitoneal injection. The vehicle group was treated with 0.9% saline. H&E and Masson staining of peritendinous adhesion revealed fewer adhesion in the parecoxib treatment group than in the vehicle group on Day 14 after modeling (**Figure** **7**a,b). The parecoxib treatment group had a decreased adhesion area and adhesion grading scale on Days 14 after modeling (Figure 7c,d). Parecoxib also improved the ROM on Days 14 after modeling (Figure 7e,f). Parecoxib reduced the mRNA expression of *Col1a1, Col3a1* and PGE2 levels in peritendinous adhesion tissues compared with that in the vehicle group on Day 14 after modeling (Figure 7g–i). However, the repaired tendons treated with parecoxib had a lower maximum load and stiffness than those in the vehicle group on Day 14 after surgery (Figure 7j,k). To determine the ability of COX2 inhibitors to decrease peritendinous adhesion, we compared the Total Active Motion (TAM) of peritendinous adhesion between patients treated with the COX2 inhibitor celecoxib and untreated patients. The results showed that celecoxib treatment improved the TAM at 6 weeks, 3 months, 6 months, and 12 months after treatment (Figure 7l). These experiments revealed that inhibited COX2 can reduce PGE2 levels and adhesion formation.

These results suggested that the COX2 inhibitor parecoxib reduced mouse PAF but impaired the biomechanical properties of repaired tendons.

### 2.6 PGE2 Treatment Improves the Biomechanical Properties of Repaired Tendons but Increases PAF

**Figure 8:**
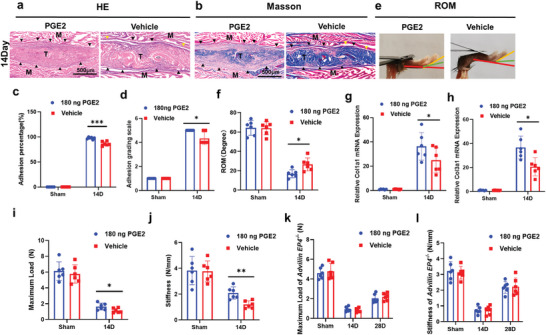
PGE2 exacerbated PAF but improved the biomechanical properties of repaired tendons. C57BL/6J WT mice were treated with PGE2 (180 ng per week) or vehicle (saline). a,b) Representative images of H&E (a) and Masson (b) staining of peritendinous tissues from WT mice treated with PGE2 or vehicle at 14 days after modeling. Yellow arrowheads indicate the space between the tendon and surrounding tissues. Black arrowheads indicate the space occupied by adhesion tissues. Scale bar, 500 µm. c,d) Adhesion percentage (c) and adhesion grading scale (d) of the peritendinous tissues from WT mice treated with PGE2 or vehicle at 14 days after modeling. e,f) Investigation analysis (e) and quantitative analysis (f) of ROM. g,h) Relative mRNA expression of *COL1A1* (g) and *COL3A1* (h) of peritendinous tissues from WT mice treated with PGE2 or vehicle at 14 days after modeling. i,j,k,l) Maximum load (i,k) and stiffness (j,l) of repaired tendons from WT or *Advillin EP4* ^‐/‐^ mice treated with PGE2 or vehicle at 14 days after modeling. ^*^ indicates *p* < 0.05. ^**^ indicates *p* < 0.01. ^***^ indicates *p* < 0.001. *n* = 6 per group (c–l). M, muscle; T, tendon; D, day; ROM, range of motion.

A recent study revealed that a high level of PGE2 promoted tendon healing.^[^ ^27^ ^]^ However, whether PGE2 treatment increases peritendinous adhesion is unknown. Therefore, we injected PGE2 (180 ng per week) into the bilateral flexor digitorum longus tendons of C57BL/6J WT mice after tendon modeling. The mice were treated with 180 ng of PGE2 on Days 0 and 7 after modeling. The vehicle group was treated with 0.9% saline. H&E and Masson staining of peritendinous adhesion revealed more adhesion in the PGE2 treatment group than in the vehicle group on Day 14 after modeling (**Figure** **8**a,b). The PGE2 treatment group had more adhesion area and adhesion grading scale on Day 14 after modeling (Figure 8c,d). PGE2 treatment reduced the ROM on Days 14 after modeling (Figure 8e,f). PGE2 treatment increased the mRNA expression of *Col1a1* and *Col3a1* in peritendinous adhesion tissues compared with that in the vehicle group on Day 14 after modeling (Figure 8g,h). Compared with those in the vehicle group, the tendons in the PGE2 treatment group exhibited increased maximum load and stiffness on Day 14 after modeling (Figure 8i,j). However, compared with vehicle treatment, PGE2 treatment did not improve the maximum load or stiffness of the repaired tendons in the *Advillin EP4^−/−^* mice. These findings were consistent with previous results, *Advillin EP4^−/−^* mice had higher PGE2 level but the biomechanical properties of repair tendons were poorer after modeling, suggesting that PGE2 played a role in tendon repair through EP4 in sensory nerves (Figure 8k,i).

These results suggested that PGE2 treatment improved the biomechanical properties of repaired tendons by activating EP4 in sensory nerves but increased peritendinous adhesion.

## 3 Discussion

PAF is a daunting challenge for hand surgeons.^[^ ^28^ ^]^ To elucidate the mechanisms of PAF to contribute to drug therapy, we performed scRNA‐seq of human and mouse peritendinous adhesion tissues. The results of scRNA‐seq revealed that COX2 was mostly expressed in myeloid cells (including macrophages and neutrophils) and fibroblasts. EP1, EP2, and EP3 were rarely expressed in peritendinous adhesion. EP4 was mostly expressed in macrophages and fibroblasts. By generating eight different transgenic mice, we identified myeloid cells and sensory nerves as the potential origin and effector cells of PGE2, respectively, and excluded the involvement of myofibroblasts and stem cells in COX2/PGE2/EP4 pathway‐mediated PAF. The inhibition of PGE2 reduced PAF but impaired tendon biomechanical properties. In addition, PGE2 treatment improved the biomechanical properties of repaired tendons but increased peritendinous adhesion.

The potential origin of PGE2 in myeloid cells has been reported.^[^ ^29^ ^]^ In this study of PAF, based on our scRNA‐seq analysis of human PAF specimens, PGE2 was highly expressed in myeloid cells (including macrophages, neutrophils, and mast cells). To further study the in vivo function of PGE2, we generated a peritendinous mouse model, and scRNA‐seq analysis of mouse peritendinous adhesion specimens was performed. As results, the COX2 expression in macrophages and neutrophiles in human peritendinous adhesion tissues showed the same trend as that in mouse peritendinous adhesion tissues. Subsequently, we deleted COX2 in both macrophages and neutrophils, by generating transgenic *Lysm‐cre: COX2^flox/flox^* mice, and the significant anti‐adhesion effect was revealed in this mouse model. This finding indicated that myeloid cells were the crucial origin of PGE2 during PAF. COX2 was also expressed in mouse peritendinous adhesion fibroblasts, and the deletion of COX2 in fibroblasts reduced PAF. However, the expression of COX2 in fibroblasts was detected only in mouse rather than human peritendinous adhesion tissues via scRNA‐seq analysis. Therefore, there is no clinical value of using fibroblasts as potential targets. Furthermore, the strength of the healing tendon decreased in the myeloid cell specific transgenic mice. Recent studies have shown that inhibiting PGE2 with COX2 inhibitors can reduce pain and inflammation but impair tendon repair.^[^ ^25^, ^26^ ^]^ In this clinical study, we revealed that a COX2 inhibitor could improve the TAM in patients. However, the COX2 inhibitor parecoxib decreased PGE2 levels and impaired tendon repair. These findings indicate that the use of COX2 inhibitors to prevent PAF should be performed carefully.

EP1, EP2, EP3, and EP4 are all PGE2 receptors.^[^ ^5^, ^12^ ^]^ In this study, based on our scRNA‐seq analysis of human and mouse peritendinous adhesion specimens, only EP4, rather than EP1, EP2, or EP3 was expressed in fibroblasts and macrophages. However, PAF was not significantly inhibited by the deletion of EP4 in fibroblasts or macrophages.^[^ ^8^ ^]^ Recently, deletion of EP4 in sensory nerves was found to reduce bone mass.^[^ ^24^ ^]^ Furthermore, these findings indicated that the brain was involved in mediating the COX2/PGE2/EP4 pathway and bone mass.^[^ ^24^, ^30^ ^]^ Sensory nerves were previously observed in tendons.^[^ ^14^ ^]^ Sensory nerve fibers are often identified by the expression of CGRP and participate in tendon healing.^[^ ^14^ ^]^ Tendon healing has three continuous phases: the inflammatory phase, the proliferative phase, and the remodeling phase.^[^ ^1a^ ^]^ Previous studies revealed that CGRP sensory nerves infiltrated the injury site and induced vessel permeation at the proliferative phase and the remodeling phase, eventually leading to scar formation.^[^ ^31^ ^]^ Thus, we selected sensory nerves as potential effector cells of EP4. We found that PAF was significantly prevented in the mice with deletion of EP4 in sensory nerves. We revealed that CGRP sensory nerves also participated in the inflammatory phase (the adhesion reduced at 7 days post injury in *Advillin EP4^−/−^* mice). Therefore, the CGRP sensory nerves participated in a total of three phases of tendon healing. Our results showed that CGRP sensory nerves infiltration increased peritendinous adhesion.

Other studies have suggested that PGE2 played an active role in tissues repair and regeneration.^[^ ^5^, ^27^, ^32^ ^]^ In this study, we treated peritendinous adhesion with PGE2 local injection in mouse model. These results suggested that the PGE2 local treatment promoted tendon repair but increased peritendinous adhesion. However, the deletion of EP4 in sensory nerves blocked the effect of PGE2. According to our results, PGE2 increased *Col1a1* and *Col3a1* expression, which could explain how PGE2 treatment promoted tendon repair and PAF. However, several studies have suggested that high levels of PGE2 induced tendon degeneration and decreased proliferation of tendon fibroblasts.^[^ ^33^ ^]^ Combined with our results, the exact dose of PGE2 require to promote tendon repair needs further study. Consequently, we believe that myeloid cell‐derived PGE2 can activate EP4 in sensory nerves to promote PAF and tendon repair in mouse PAF model.

Our study had several limitations. The expression of EP4 was high in T cells of human peritendinous adhesion tissues. The effects of EP4 in T cells during human PAF had not been verified. Furthermore, we only chose Day 14 post modeling as the time point, and the prognostic value of parecoxib and PGE2 is still unknown. The exact dose of PGE2 local treatment to promote tendon repair needs further study. Our study was limited to the periphery, and the role of the brain in PAF was not examined. Although several limitations exist, our results were relevant.

We conclude that myeloid cells secrete PGE2, which activates EP4 in sensory nerves to induce PAF but promotes tendon healing. The sensory nerves are affected by PGE2 and participate in PAF. PGE2 local treatment promoted tendon repair but increased peritendinous adhesion. PGE2 may be a treatment for promoting the biomechanical properties of tendons. Our findings clarified the complex role of PGE2 in PAF and tendon repair.

## 4 Experimental Section

### Single‐Cell RNA Sequencing

NovelBio BioPharm Technology Co., Ltd. performed scRNA‐Seq data analysis with the NovelBrain Cloud Analysis Platform. Filtered cells containing over 200 expressed genes and mitochondrial unique molecular identifiers (UMI) rates below 20%. Used the Seurat package (version 3.1.4, https://satijalab.org/seurat/) for cell normalization and regression. Used the fastMNN function (k = 10, d = 50, approximate = TRUE) in the R package scran (v1.12.1) to apply the mutual nearest neighbor method to correct for batch effects among samples. Finally, got the unsupervised cell cluster result by using the graph‐based cluster method (resolution = 0.8).

The human samples were got from patients who had peritendinous adhesion for 3, 10, and 90 days. The mouse samples were got from mouse which got modeling for 7, 10, 14, and 28 days. Only scrape off the peritendinous adhesion tissues without tendon tissues.

### Human Subjects

Human peritendinous adhesion tissues were surgically resected from flexor tendon tenolysis patients at Wuxi Ninth People's Hospital (Wuxi, China). All patients were collected at orthopedics of Wuxi Ninth People's Hospital. The study was approved by The Ethical Committee of Wuxi Ninth People's Hospital and all patients signed informed consent. The number was KT201803. The peritendinous granulation adhesion tissues were scarped around tendon trauma without tendon tissues.

40 patients with peritendinous adhesion were collected at Wuxi Ninth People's Hospital (Wuxi, China) during 2021–2022, randomly selected 20 patients of them accepted celecoxib treatment orally (200 mg per day) for two weeks. The remaining 20 patients with nonuse were taken as control. Evaluated the Total Active Motion (TAM) at 6 weeks, 3 months, 6 months and 12 months after treatment.

### Mouse Subjects

All the mice experiments accorded with the guidelines published by NIH and Shanghai Sixth People's Hospital Internal Review Board (Shanghai, China). All C57BL/6J mice were purchased from the animal facility of Shanghai Sixth People's Hospital. Constructing the mouse peritendinous adhesion model, narcotizing the eight‐week‐old male mice by 3% pentobarbital sodium. Then disinfected the skin in the right hind‐paw by 75% ethanol. Sectioning the skin and exposing the flexor digitorum longus tendon. Then cut off the tendon and repaired it by modified Kessler pattern, using 8‐0 sutures. Finally, closing the wound with 6‐0 sutures (Figure S9, Supporting Information). All the mice were treated as equal and allowed to move freely in the cage. The Sham surgery followed the same incision procedure, but the deep fascia and tendon were left intact.

Heterozygous *Lysm‐cre*, heterozygous *S100a4‐cre*, heterozygous *α‐SMA‐cre*, and *COX2^flox/flox^* mice were purchased from Shanghai Southern model organisms. Heterozygous *Advillin‐cre^ERT^* ^2^ mice were purchased from Jiangsu GemPharmatech Company. Heterozygous *Prrx1‐cre* and *EP4^flox/flox^* mice were donated from Bio‐X Institutes of Shanghai Jiao Tong University. Then *Lysm‐cre:COX2^flox/flox^* (*Lysm COX2^−/−^*) mice were generated by crossing heterozygous *Lysm‐cre* mice with *COX2^flox/flox^* (*COX2^−/−^*) mice. *S100a4‐cre:COX2^flox/flox^* (*S100a4 COX2^−/−^*) mice were generated by crossing heterozygous *S100a4‐cre* mice with *COX2^flox/flox^* (*COX2^f/f^*) mice. *α‐SMA‐cre:COX2^flox/flox^* (*α‐SMA COX2^−/−^*) mice were generated by crossing heterozygous *α‐SMA‐cre* mice with *COX2^flox/flox^* (*COX2^−/−^*) mice. *Advillin‐cre^ERT2^:EP4^flox/flox^* (*Advillin EP4^−/−^*) mice were generated by crossing heterozygous *Advillin‐cre^ERT2^* mice with *EP4^flox/flox^* (*EP4^f/f^*) mice, before modeling, intraperitoneal injection of 100 mg kg^−1^ body weight tamoxifen or vehicle was performed three times per week until the mice were euthanized. *S100a4‐cre:EP4^lox/flox^* (*S100a4 EP4^−/−^*) mice were generated by crossing heterozygous *S100a4‐cre* mice with *EP4^flox/flox^* (*EP4^f/f^*) mice. *Lysm‐creEP4^lox/flox^* (*Lysm EP4^−/−^*) mice were generated by crossing heterozygous *Lysm‐cre* mice with *EP4^flox/flox^* (*COX2^f/f^*) mice. *Prrx1‐cre:EP4^flox/flox^* (*Prrx1 EP4^f/f^*) mice were generated by crossing heterozygous *Prrx1‐cre* mice with *EP4^flox/flox^* (*EP4^f/f^*) mice. *α‐SMA‐cre:EP4^flox/flox^* (*α‐SMA EP4^−/−^*) mice were generated by crossing heterozygous *α‐SMA‐cre* mice with *EP4^flox/flox^* (*EP4^f/f^*) mice. The peritendinous adhesion model was established in 8‐week‐old male *Lysm COX2^−/−^, S100a4 COX2^−/−^, α‐SMA COX2^−/−^, Advillin EP4^−/−^, S100a4 EP4^−/^, Lysm EP4^−/−^, Prrx1 EP4^−/−^, α‐SMA EP4^−/−^*, *COX2^f/f^* and *EP4^f/f^* mice (n = 6 per group), which were euthanized after 7, 10, 14, or 28 days for further analysis.

For the treatment experiments, C57BL/6J wild‐type mice got intraperitoneal injection of 16 mg kg^−1^ parecoxib (Merck, 32 152) per day after modeling, and the vehicle group got intraperitoneal injection of saline as replacement, which were euthanized after 14 days for further analysis.

For the PGE2 experiments, C57BL/6J wild‐type and *Advillin EP4* ^‐/‐^ mice injected 180 ng PGE2 (Merck, 900117P) into bilateral flexor digitorum longus tendon injection at Days 0 and 7 after modeling and the vehicle group injected saline as replacement, which were euthanized after 14 or 28 days for further analysis.

### Histological Staining andImmunofluorescence Analysis

The mice were euthanized by inhalation of carbon dioxide. Then, the mice were fixed with 4% paraformaldehyde via perfusion through the left ventricle for 5 min. Then collected the mice feet and soaked in 4% paraformaldehyde 1 day at 4 °C. Then decalcified mice feet by 20% EDTA (buffered with phosphate‐buffered saline [PBS], pH 7.4) for 6 days. Dehydrated specimens by 30% sucrose (Sigma‒Aldrich, S9378) for 1 day at 4 °C. Finally, embedded the specimens by OCT at −20 °C. 10‐µm sagittal sections were cut and subsequently subjected to H&E and Masson staining. We performed immunofluorescence staining of peritendinous adhesion. The frozen sections were washed three times PBS. We performed immunofluorescence staining of PAF sections. Washing sections 3 times with PBS. After blocking endogenous peroxidase activity and nonspecific sites, the tissue sections were incubated with primary antibodies against CD68 (1:200), COX2 (1:200), EP4 (1:100), and CGRP (1:300), for 1 day at 4 °C. Subsequently, secondary antibodies were added and incubated with the sections at room temperature for 1 h. Then covered the sections by DAPI (Life Technologies, P36935). Images were captured with a fluorescent microscope (Leica, DM6). We used ImageJ software to analyze the results.

### Antibodies

COX2, Abcam, ab179800, 1:200

CD68, Abcam, ab53444, 1:200

EP4, Thermo Fisher Scientific, BS‐8538R, 1:100

CGRP, Abcam, ab81887, 1:300

Alexa Fluor 488, Abcam, ab150077, 1:1000

Alexa Fluor 594, Abcam, ab150160, 1:1000

Alexa Fluor 594, Abcam, ab150116, 1:1000

### Analysis of Peritendinous Adhesion Percentage and Adhesion Grading Scale

Evaluate the HE staining of peritendinous adhesion and calculate the percentage of adhesion aera account for all area by Image J. Grade 1, the percentage of adhesion aera for all area < 5%. Grade 2, 5%−30%. Grade 3, 30%‐ 60%. Grade 4, 60%‐ 90%. Grade 5, >90%.

### Enzyme‐Linked Immunosorbent Assay (ELISA) of Peritendinous Adhesion PGE2 Levels

The concentration of PGE2 level in mice peritendinous adhesion tissues around the repaired tendon was detected by the PGE2 ELISA Development kit (Solarbio, SEKM‐0173) according to the vendor's protocol.

### RNA Extraction and RT‐qPCR

Isolated RNA from mice peritendinous adhesion tissues by using RNAprep Pure Micro Kit (DP420, TIANGEN BIOTECH (BEIJING) CO., LTD), then using HiScript III RT SuperMix for QPCR (Vazyme, R323‐01) to perform cDNA synthesis. All steps were according to the manufacturer's protocol. Then performed reactions in triplicate in 384‐well plate format. Performed RT‐qPCR for tissues by using ChamQ Universal SYBR qPCR Master Mix (Vazyme, Q711‐02) with the following primers (all Sangon Biotech): *GAPDH* (Forward AGGTCGGTGTGAACGGATTTG, Reversed TGTAGACCATGT AGTTGAGGTCA), *COL1A1* (Forward GCTCCTCTTAGGGGCCACT, Reversed CCACGTCTCACCATTGGGG), *COL3A1* (Forward CTGTAACATGGAAACTGGGGAAA, Reversed CCATAGCTG AACTGAAAACCACC), *COX2* (Forward TTCAACACACTCTATCACTGGC, Reversed AGAAGCGTTTGCGGTACTCAT), *EP4* (Forward ACCATTCCTAGATCGAACCGT, Reversed CACCACCCCGAAGATGAACAT). Amplified the samples on an ABI 7900HT FAST PCR system (Applied Biosystems, ThermoFisher Scientific) and analyzed the data with ThermoFisher Connect cloud qPCR analysis software (ThermoFisher Scientific). Then used GAPDH for normalization to quantify the 2^−Δ Δ Ct^. Estimated the amount of target mRNA in samples and expression calculated relative to average mRNA expression by using 2^−Δ Δ Ct^ quantification method. Using GAPDH for normalization to quantify the 2^−Δ Δ Ct^.

### Assessment of Gliding Function

The skin of the hind limb that was amputated at the knee level was removed down to the ankle (n = 6 per group). The proximal terminal FDL was exposed and secured using cyanoacrylate between 2 pieces of tape. First, the tibia was stabilized by an alligator clip. Then, the FDL was pulled with 19 g weights, and associated digital images were captured to investigate the ROM of the metatarsophalangeal joint. Complete flexion was defined according to that of the digits with uninjured tendons loaded with 19 g. Finally, gliding resistance was calculated based on the correspondence of the ROM with loads.

### Biomechanical Testing

To evaluate the biomechanical properties of tendon, we calculated the maximum load and stiffness of the repaired tendons at sham and Days 14 and 28 post‐modeling. by using a Dynamic Mechanical Analyzer Q800 (TA Instruments). Preparing the specimens by amputation of the tibia at the ankle. Stabilizing the terminals of the repaired tendon to the opposing ends of force gauges. Then, pulling proximal terminal in tension at a rate of 30 mm per min until rupture to obtain a force‒displacement curve. The maximum load was documented automatically by the rheometer and stiffness was calculated as the slope of the linear region of the force‒displacement curve.

### Analysis of Total Active Motion (TAM)

The Total Active Motion (TAM) was described by the American Society for Surgery of the Hand (sum of the degrees of active MCP, PIP, and DIP joint flexion less the degrees from full extension). Take the contralateral finger (uninjured) as the normal TAM, the TAM of the injured finger was divided by the normal TAM. The result was the percentage of normal TAM. MCP joint, Metacarpophalangeal Joint. PIP joint, Proximal Interphalangeal Joint. DIP joint, Distal Interphalangeal Joint.

### Statistical Analysis

ImageJ, GraphPad Prism 8.0, and SPSS software (version 10.0, IBM Corp, Armonk, New York, USA) were used for statistical analysis. The results were presented as the mean ± SEM. Paired 2‐tailed *t*‐tests were used for comparisons between two groups. A significant difference was considered when *p* < 0.05.

## Conflict of Interest

The authors declare no conflict of interest.

## Author Contributions

X.Z., Y.X., Z.T., and Y.Z. contributed equally to this work. Study design: X. Y., S.L., and X.Z. Immunofluorescent staining: Y.X. Study conduction and data collection and analysis: X.Z., X.C., Y.Z., Z.T., Y.L., S.W., and T.Z. Manuscript preparation: X.Z. These authors contributed equally: X.Z., Y.X., Z.T., and Y.Z. Clinical samples and patients collection: X.L., W.Y., and J.T.

## Supporting information

Supporting Information

## Data Availability

The data that support the findings of this study are available from the corresponding author upon reasonable request.
