# Supplementary material for: Myeloid Cells and Sensory Nerves Mediate Peritendinous Adhesion Formation via Prostaglandin E2
Source: Adv Sci (Weinh). 2024 Aug 29;11(40):2405367. doi: 10.1002/advs.202405367 (PMC11516151; doi:10.1002/advs.202405367)
Supplement: Supplementary file 1 — Supporting Information [file ADVS-11-2405367-s001.docx]

Supporting Information

**Myeloid cells and sensory nerves mediate peritendinous adhesion formation via prostaglandin E2**

*Xinshu Zhang, Yao Xiao, Zaijin Tao, Yizhe Zhang, Xuan Cheng, Xuanzhe Liu, Yanhao Li, Weiguang Yin, Jian Tian, Shuo Wang, Tianyi Zhang, Xiao Yang, Shen Liu**


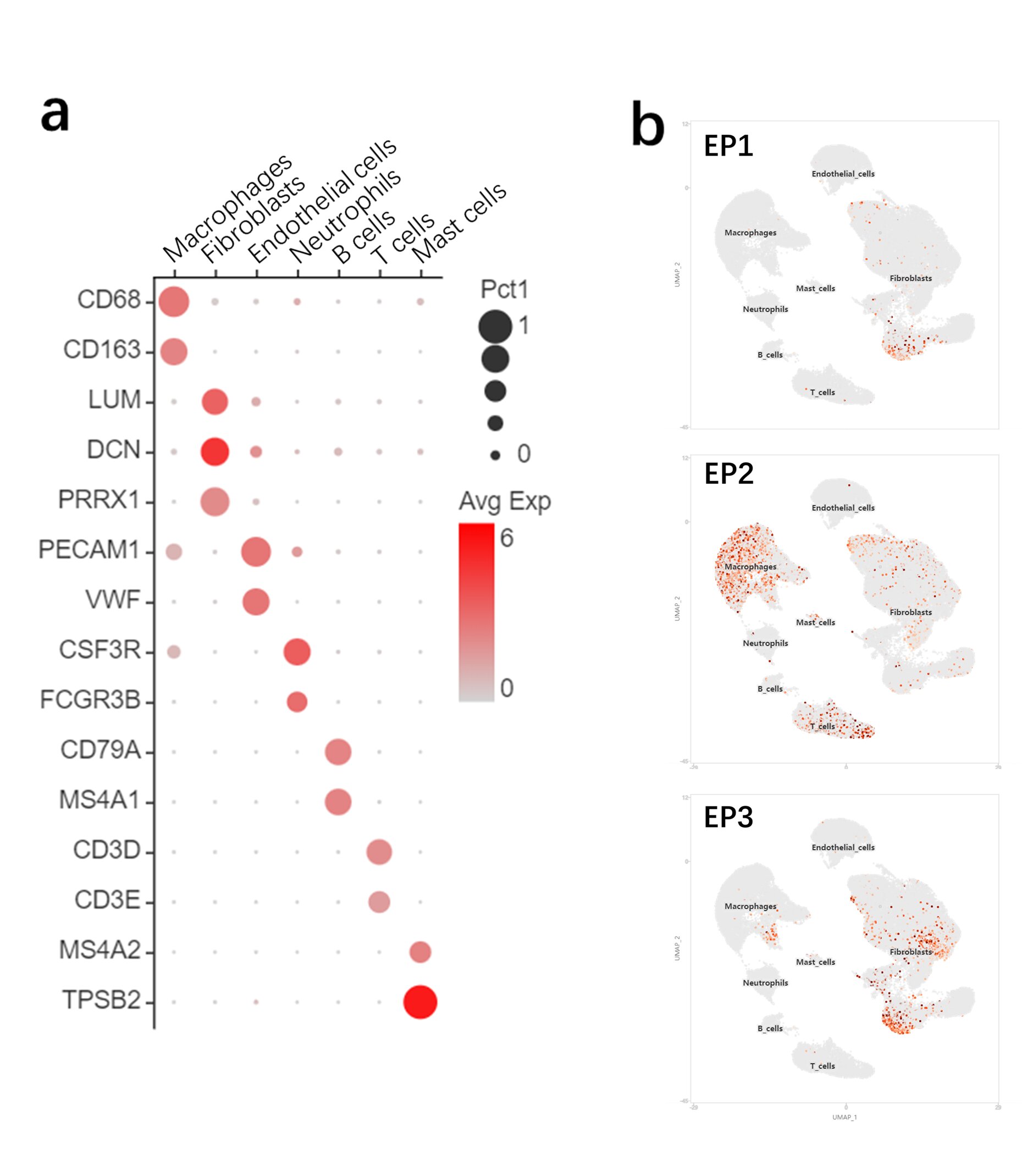


**Fig S1 a** Dot plots of marker genes of each cluster in human peritendinous adhesion tissues. **b** UMAP plots showing the expression of EP1, EP2, and EP3 of each cluster of human peritendinous adhesion tissues. UMAP, uniform manifold approximation and projection.


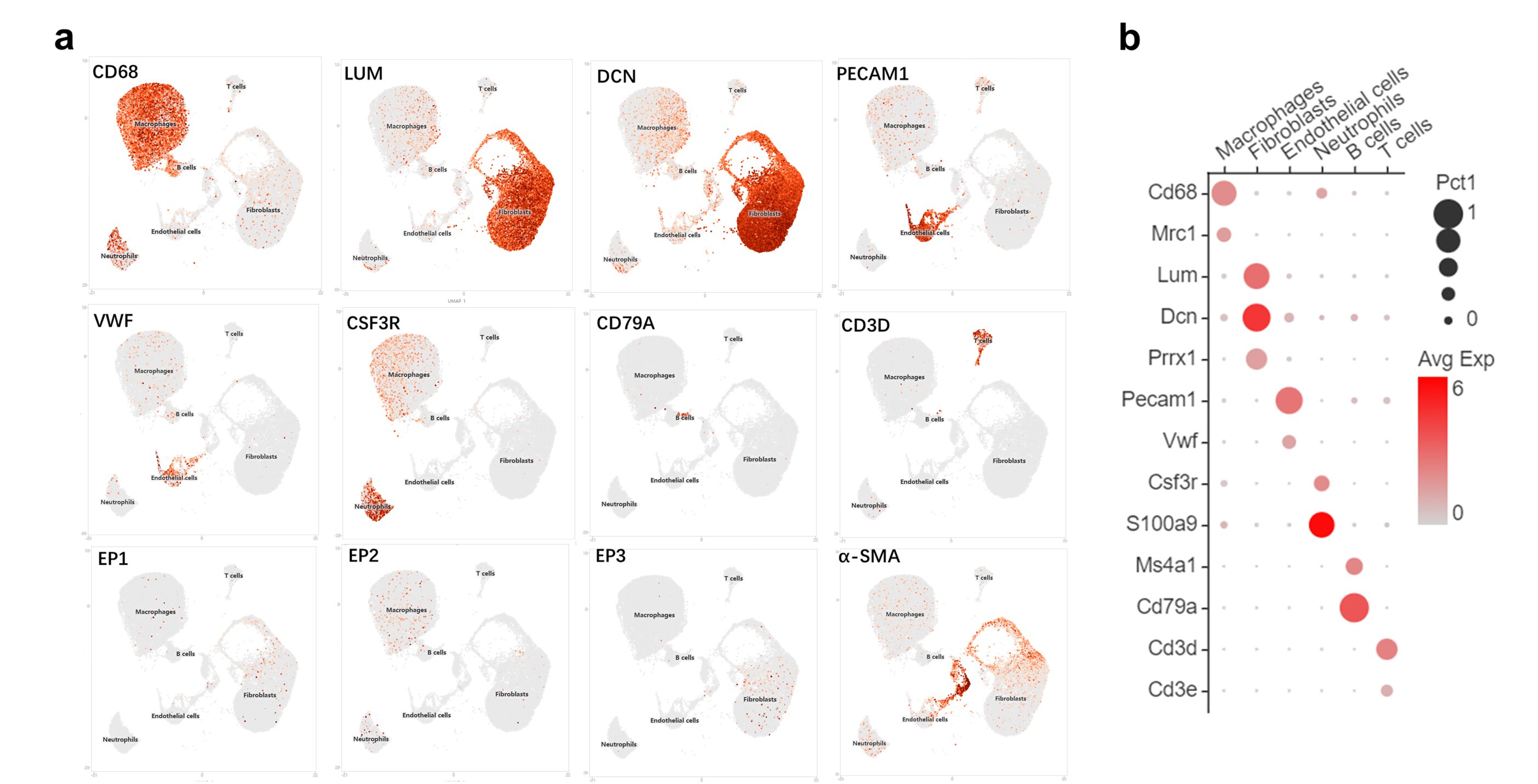


**Fig S2 a** UMAP plots showing the expression of marker genes and EP1, EP2, and EP3 in each cluster of mouse peritendinous adhesion tissues. **b** Dot plots of marker genes of each cluster. UMAP, uniform manifold approximation and projection.


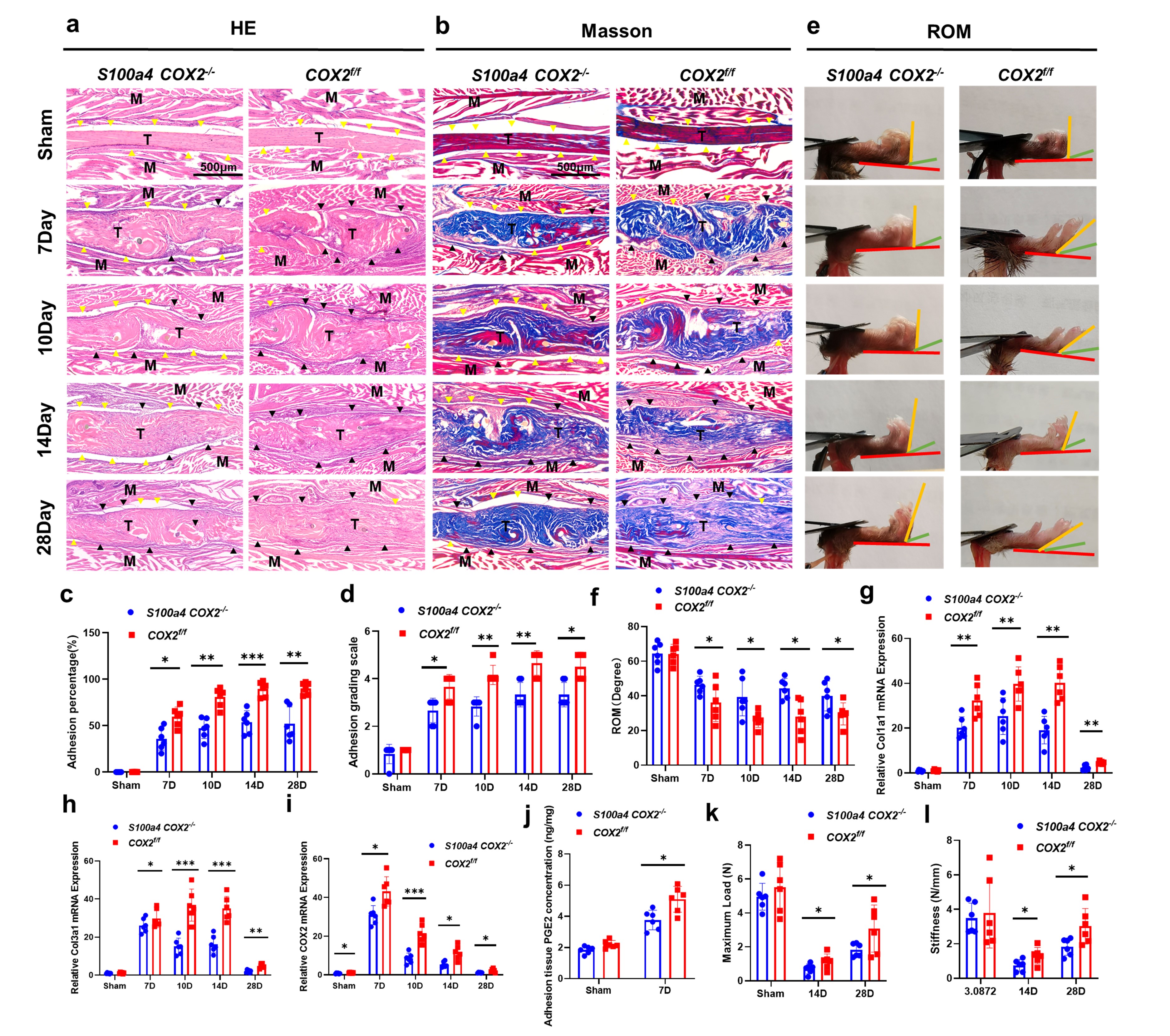


**Fig S3 Deletion of COX2 in S100a4^+^ cells attenuated PAF but impaired the biomechanical properties of repaired tendons.** *S100a4-cre: COX2^flox/flox^* (*S100a4 COX2^-/-^*) mice were generated by breeding *S100a4-cre* mice with *COX2^flox/flox^* (*COX2^f/f^*) mice. **a, b** Representative images of H&E (**a**) and Masson (**b**) staining of the peritendinous tissues from *S100a4 COX2^-/-^* and *COX2^f/f^* mice at 7, 10, 14 and 28 days after modeling. Yellow arrowheads indicate the space between the tendon and surrounding tissues. Black arrowheads indicate the space occupied by adhesion tissues. Scale bar, 500 μm. **c, d** Adhesion percentage (**c**) and adhesion grading scale (**d**) of the peritendinous tissues from *S100a4 COX2^-/-^* and *COX2^f/f^* mice at 7, 10, 14 and 28 days after modeling. **e, f** Investigation analysis (**e**) and quantitative analysis (**f**) of ROM. **g, h, i** Relative mRNA expression of *COL1A1* (**g**), *COL3A1* (**h**) and *COX2* (**i**) of the peritendinous tissues from *S100a4 COX2^-/-^* and *COX2^f/f^* mice at 7, 10, 14 and 28 days after modeling. **j** PGE2 levels (ng PGE2/mg protein) in peritendinous tissues from *S100a4 COX2^-/-^* and *COX2^f/f^* mice at 7 days after modeling. **k, l** Maximum load (**k**) and stiffness (**l**) of repaired tendons from *S100a4 COX2^-/-^* and *COX2^f/f^* mice at 14 and 28 days after modeling. * indicates *P* < 0.05. ** indicates *P* < 0.01. *** indicates *P* < 0.001. *n*=6 per group (**c-l**). M, muscle; T, tendon; D, day; ROM, range of motion


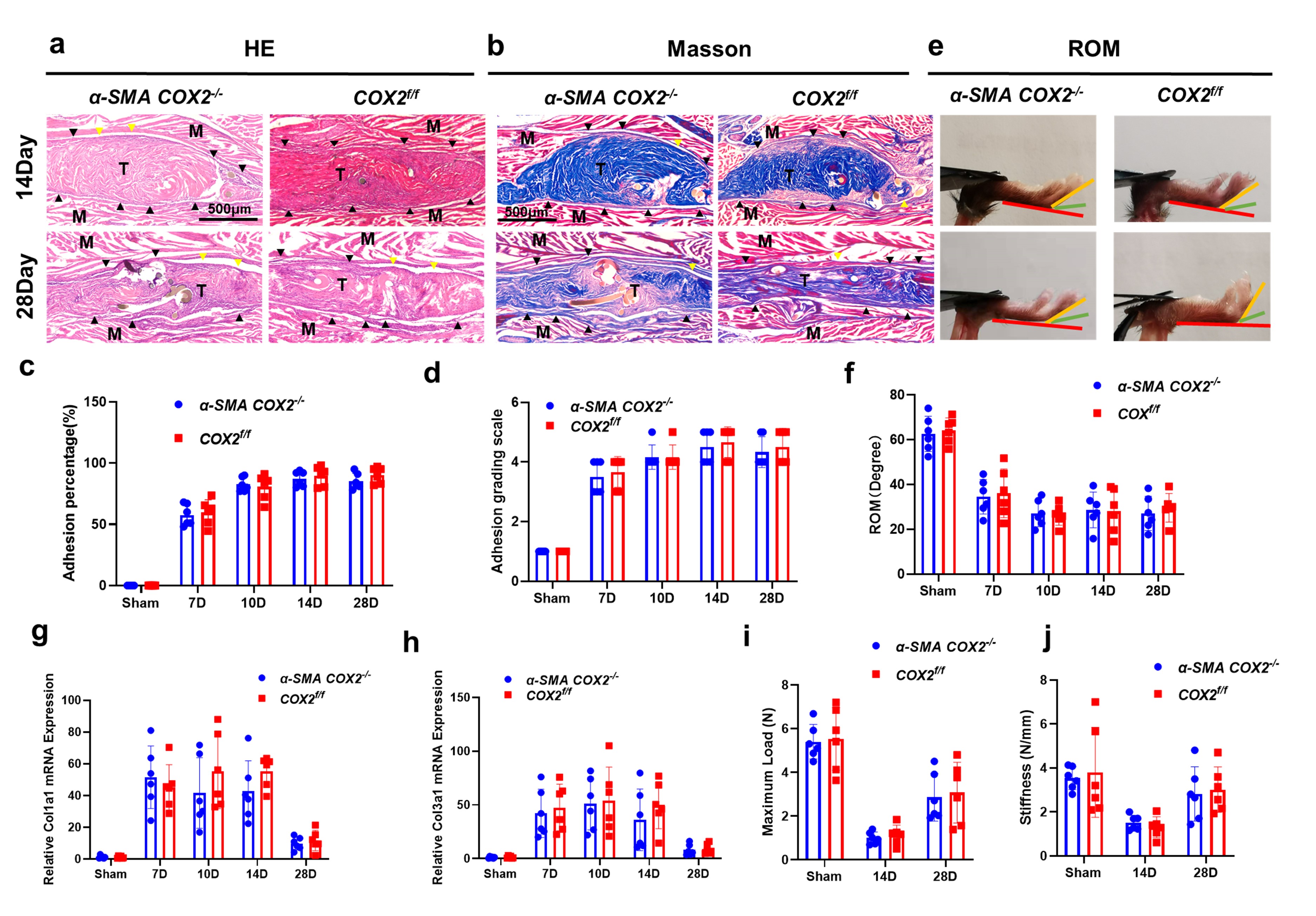


**Fig S4 Deletion of COX2 in** **α-SMA^+^ cells did not affect PAF.**

*α-SMA-cre: COX2^flox/flox^* (*α-SMA COX2^-/-^*) mice were generated by crossing *α-SMA-cre* mice with *COX2^flox/flox^* (*COX2^f/f^*) mice. **a, b** Representative images of H&E (**a**) and Masson (**b**) staining of the peritendinous tissues from *α-SMA COX2^-/-^* and *COX2^f/f^* mice at 14 and 28 days after modeling. Yellow arrowheads indicate the space between the tendon and surrounding tissues. Black arrowheads indicate the space occupied by adhesion tissues. Scale bar, 500 μm. **c, d** Adhesion percentage (**c**) and adhesion grading scale (**d**) of the peritendinous tissues from *α-SMA COX2^-/-^* and *COX2^f/f^* mice at 14 and 28 days after modeling. **e, f** Investigation analysis (**e**) and quantitative analysis (**f**) of ROM. **g, h** Relative mRNA expression of *COL1A1* (**g**) and *COL3A1* (**h**) of the peritendinous tissues from *α-SMA COX2^-/-^* and *COX2^f/f^* mice at 7, 10, 14 and 28 days after modeling. **i, j** Maximum load (**i**) and stiffness (**j**) of repaired tendons from *α-SMA COX2^-/-^* and *COX2^f/f^* mice at 14 and 28 days after modeling. * indicates *P* < 0.05. ** indicates *P* < 0.01. *** indicates *P* < 0.001. *n*=6 per group (**c- j**). M, muscle; T, tendon; D, day; ROM, range of motion


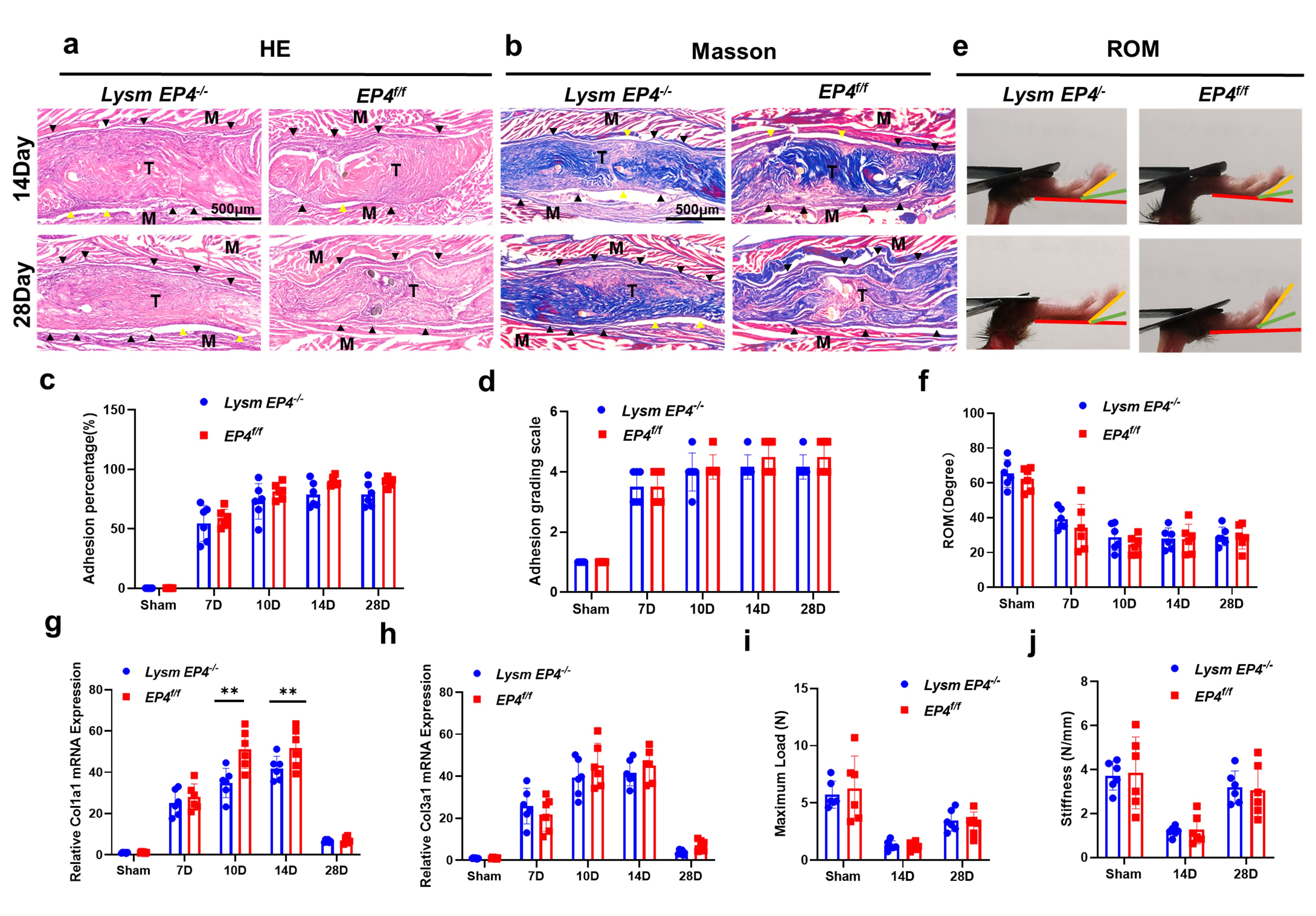


**Fig S5 Deletion of EP4 in Lysm^+^ cells did not affect PAF.**

*Lysm-cre: EP4^flox/flox^* (*Lysm EP4^-/-^*) mice were generated by crossing *Lysm-cre* mice with *EP4^flox/flox^* (*EP4^f/f^*) mice. **a, b** Representative images of H&E (**a**) and Masson (**b**) staining of the peritendinous tissues from *Lysm EP4^-/-^* and *EP4^f/f^* mice at 14 and 28 days after modeling. Yellow arrowheads indicate the space between the tendon and surrounding tissues. Black arrowheads indicate the space occupied by adhesion tissues. Scale bar, 500 μm. **c, d** Adhesion percentage (**c**) and adhesion grading scale (**d**) of the peritendinous tissues from *Lysm EP4^-/-^* and *EP4^f/f^* mice at 14 and 28 days after modeling. **e, f** Investigation analysis (**e**) and quantitative analysis (**f**) of ROM. **g, h** Relative mRNA expression of *COL1A1* (**g**) and *COL3A1* (**h**) of the peritendinous tissues from *Lysm EP4^-/-^* and *EP4^f/f^* mice at 7, 10, 14 and 28 days after modeling. **i, j** Maximum load (**i**) and stiffness (**j**) of repaired tendons from *Lysm EP4^-/-^* and *EP4^f/f^* mice at 14 and 28 days after modeling. * indicates *P* < 0.05. ** indicates *P* < 0.01. *n*=6 per group (**c- j**). M, muscle; T, tendon; D, day; ROM, range of motion


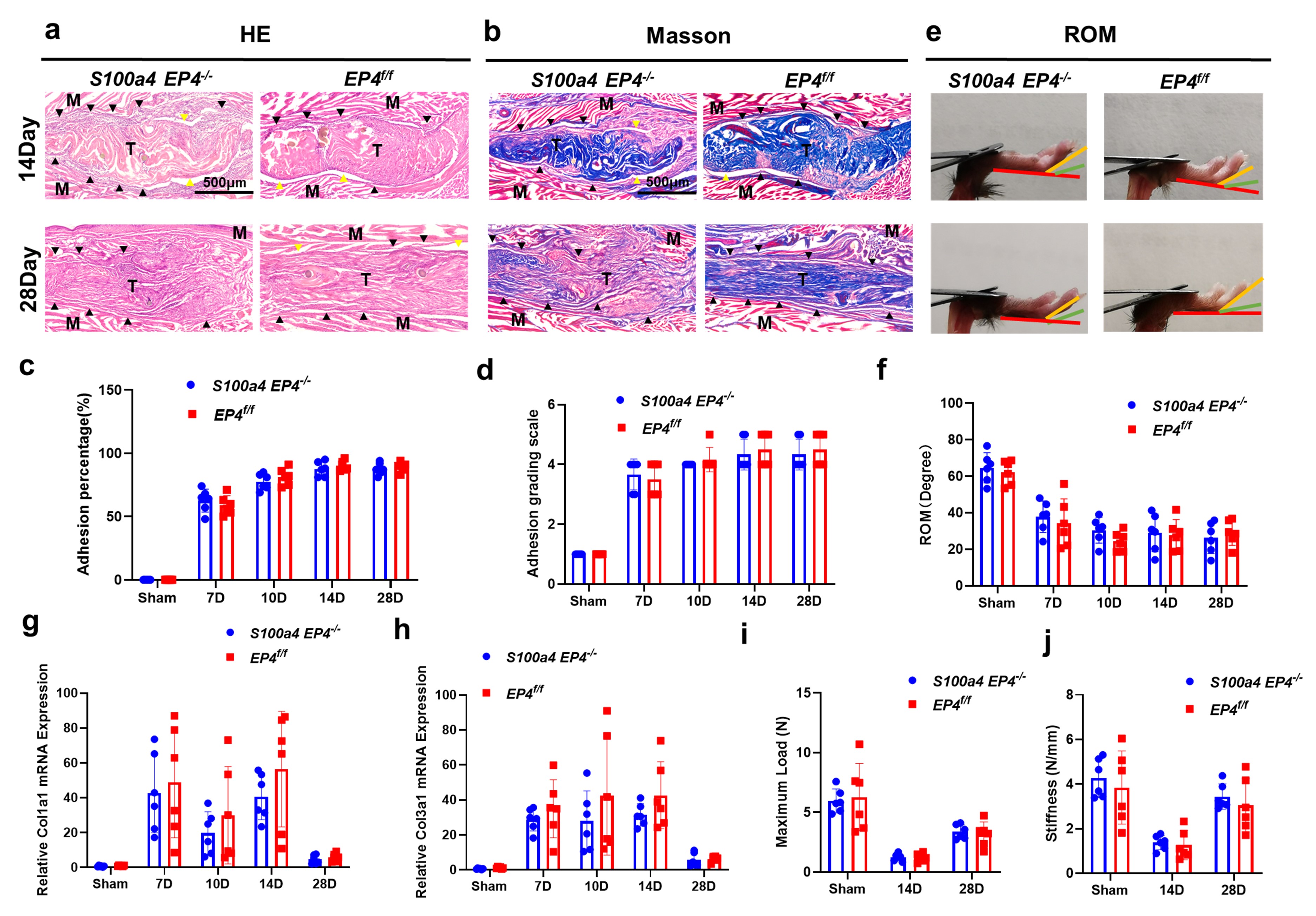


**Fig S6 Deletion of EP4 in S100a4^+^ cells did not affect PAF.** *S100a4-cre: EP4^flox/flox^* (*S100a4 EP4^-/-^*) mice were generated by crossing *S100a4-cre* mice with *EP4^flox/flox^* (*EP4^f/f^*) mice. **a, b** Representative images of H&E (**a**) and Masson (**b**) staining of the peritendinous tissues from *S100a4 EP4^-/-^* and *EP4^f/f^* mice at 14 and 28 days after modeling. Yellow arrowheads indicate the space between the tendon and surrounding tissues. Black arrowheads indicate the space occupied by adhesion tissues. Scale bar, 500 μm. **c, d** Adhesion percentage (**c**) and adhesion grading scale (**d**) of the peritendinous tissue from *S100a4 EP4^-/-^* and *EP4^f/f^* mice at 14 and 28 days after modeling. **e, f** Investigation analysis (**e**) and quantitative analysis (**f**) of ROM. **g, h** Relative mRNA expression of *COL1A1* (**g**) and *COL3A1* (**h**) of the peritendinous tissues from *S100a4 EP4^-/-^* and *EP4^f/f^* mice at 7, 10, 14 and 28 days after modeling. **i, j** Maximum load (**i**) and stiffness (**j**) of repaired tendons from *S100a4 EP4^-/-^* and *EP4^f/f^* mice at 14 and 28 days after modeling. *n*=6 per group (**c- j**). M, muscle; T, tendon; D, day; ROM, range of motion


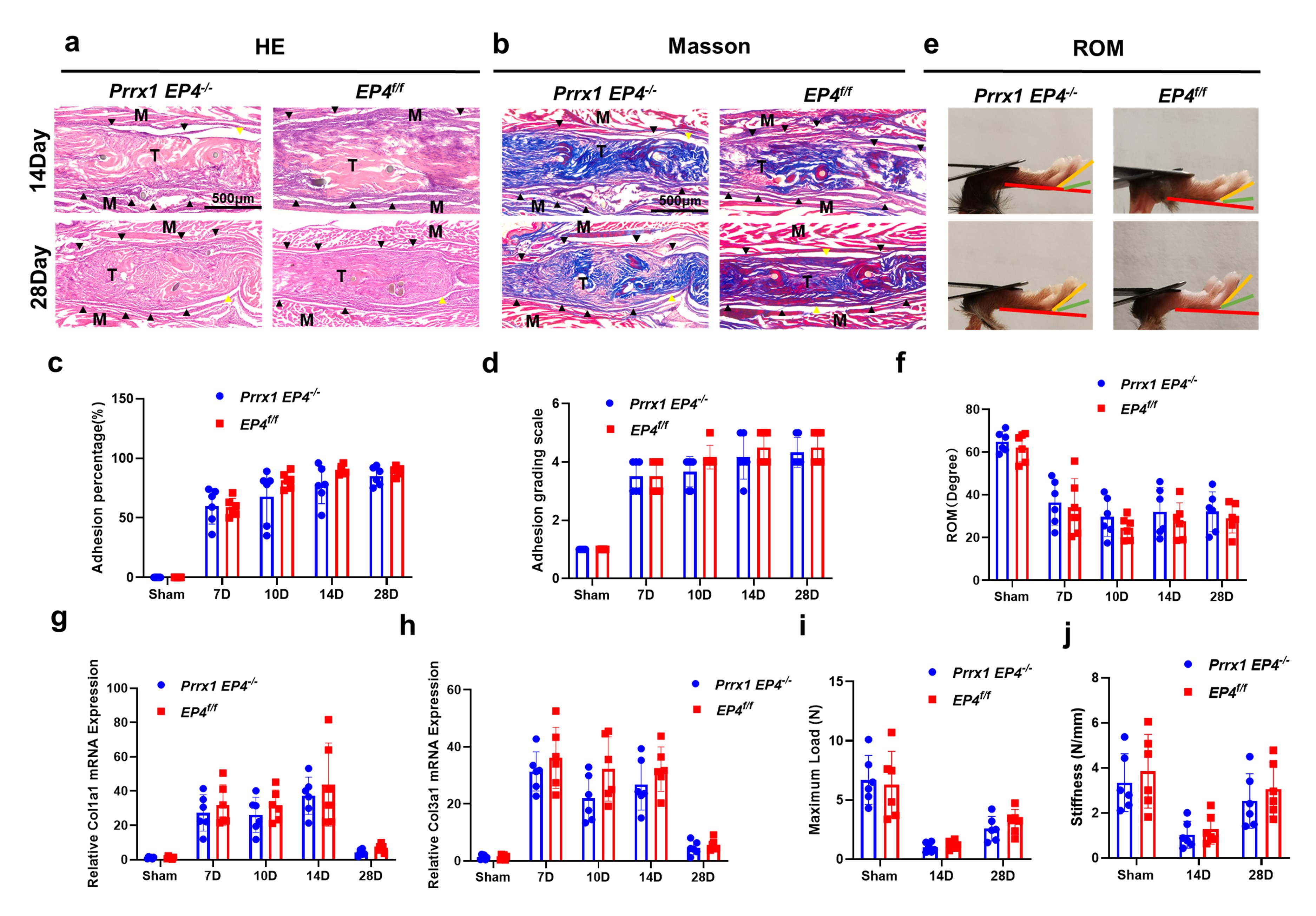


**Fig S7 Deletion of EP4 in Prrx1^+^ cells did not affect PAF.**

*Prrx1-cre: EP4^flox/flox^* (*Prrx1 EP4^-/-^*) mice were generated by crossing *Prrx1-cre* mice with *EP4^flox/flox^* (*EP4^f/f^*) mice. **a, b** Representative images of H&E (**a**) and Masson (**b**) staining of the peritendinous tissues from *Prrx1 EP4^-/-^* and *EP4^f/f^* mice at 14 and 28 days after modeling. Yellow arrowheads indicate the space between the tendon and surrounding tissues. Black arrowheads indicate the space occupied by adhesion tissues. Scale bar, 500 μm. **c, d** Adhesion percentage (**c**) and adhesion grading scale (**d**) of the peritendinous tissues from *Prrx1 EP4^-/-^* and *EP4^f/f^* mice at 14 and 28 days after modeling. **e, f** Investigation analysis (**e**) and quantitative analysis (**f**) of ROM. **g, h** Relative mRNA expression of *COL1A1* (**g**) and *COL3A1* (**h**) of the peritendinous tissues from *Prrx1 EP4^-/-^* and *EP4^f/f^* mice at 7, 10, 14 and 28 days after modeling. **i, j** Maximum load (**i**) and stiffness (**j**) of repaired tendons from *Prrx1 EP4^-/-^* and *EP4^f/f^* mice at 14 and 28 days after modeling. *n*=6 per group (**c- j**). M, muscle; T, tendon; D, day; ROM, range of motion.


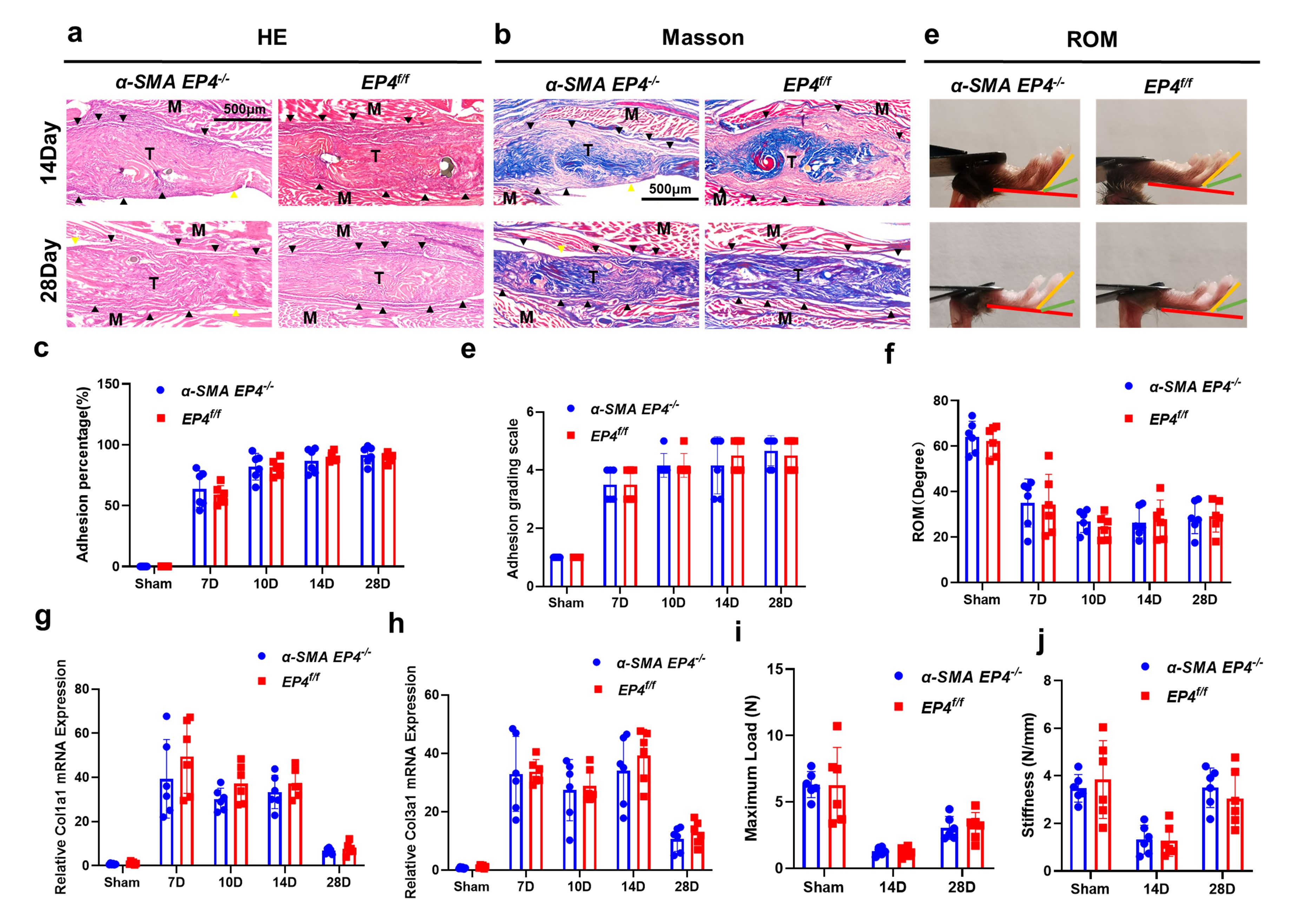


**Fig S8 Deletion of EP4 in α-SMA^+^ cells did not affect PAF.**

*α-SMA-cre: EP4^flox/flox^* (*α-SMA EP4^-/-^*) mice were generated by crossing *α-SMA-cre* mice with *EP4^flox/flox^* (*EP4^f/f^*) mice. **a, b** Representative images of H&E (**a**) and Masson (**b**) staining of the peritendinous tissues from *α-SMA EP4^-/-^* and *EP4^f/f^* mice at 14 and 28 days after modeling. Yellow arrowheads indicate the space between the tendon and surrounding tissues. Black arrowheads indicate the space occupied by adhesion tissues. Scale bar, 500 μm. **c, d** Adhesion percentage (**c**) and adhesion grading scale (**d**) of the peritendinous tissues from *α-SMA EP4^-/-^* and *EP4^f/f^* mice at 14 and 28 days after modeling. **e, f** Investigation analysis (**e**) and quantitative analysis (**f**) of ROM. **g, h** Relative mRNA expression of *COL1A1* (**g**) and *COL3A1* (**h**) of the peritendinous tissues from *α-SMA EP4^-/-^* and *EP4^f/f^* mice at 7, 10, 14 and 28 days after modeling. **i, j** Maximum load (**i**) and stiffness (**j**) of repaired tendons from *α-SMA EP4^-/-^* and *EP4^f/f^* mice at 14 and 28 days after modeling. *n*=6 per group (**c- j**). M, muscle; T, tendon; D, day; ROM, range of motion


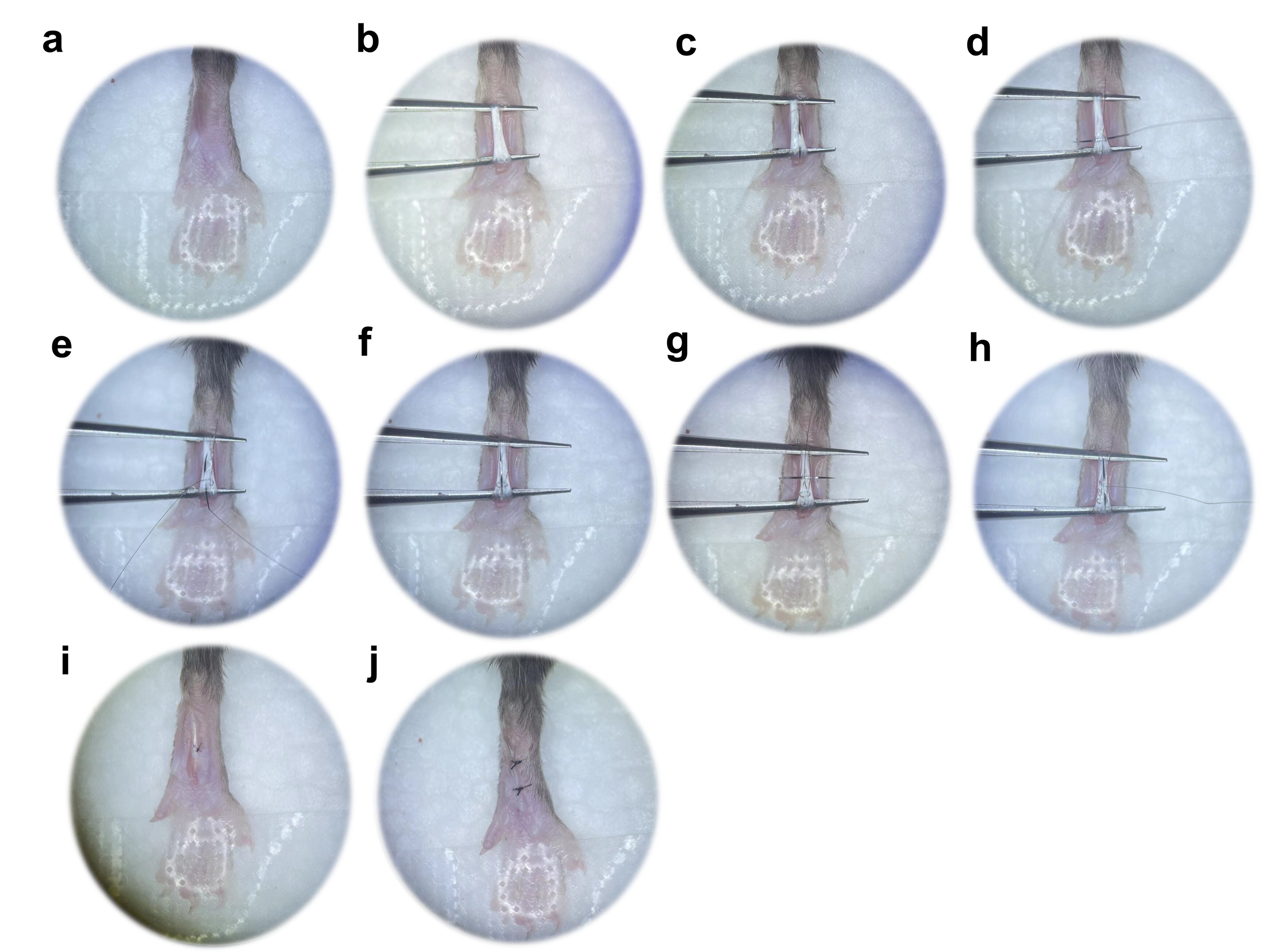


**Fig S9 Peritendinous adhesion mouse model.**

**a** The skin on the right hind paw was exposed and disinfected with 75% ethanol. **b** The skin was sectioned and the flexor digitorum longus tendon was exposed. **c-h** The tendon was sutured with a modified Kessler pattern, a total of 6 stitches, using 8-0 sutures. **i** The tendon was cut off, and the 8-0 sutures were tied. **j** The skin wound was closed with 6-0 sutures.


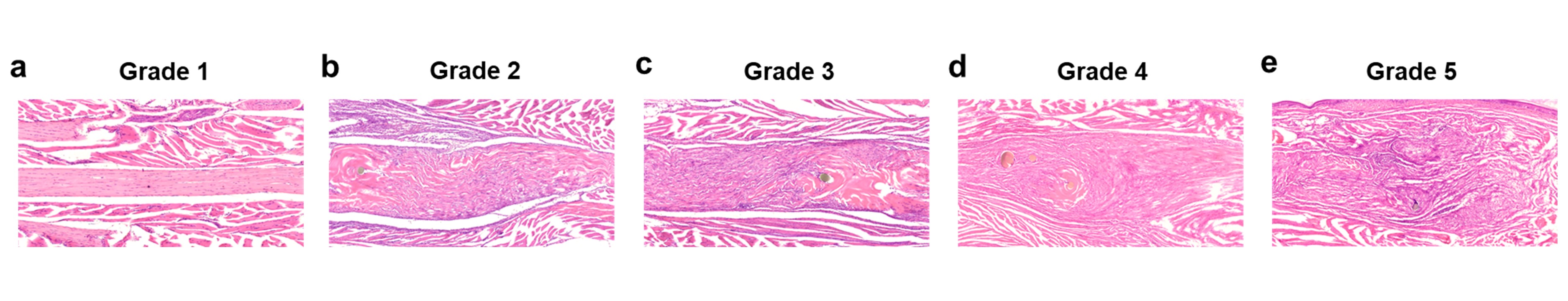


**Fig S10 Adhesion grading scale**

**a** Grade 1, the percentage of adhesion aera for all areas < 5%. **b** Grade 2, 5%-30%. **c** Grade 3, 30%- 60%. **d** Grade 4, 60%- 90%. **e** Grade 5, >90%.
